# Supplementary material for: Cancer in HIV-positive and HIV-negative adolescents and young adults in South Africa: a cross-sectional study
Source: BMJ Open. 2021 Oct 17;11(10):e043941. doi: 10.1136/bmjopen-2020-043941 (PMC8524277; doi:10.1136/bmjopen-2020-043941)
Supplement: Supplementary data [file bmjopen-2020-043941supp001.pdf]

Online Supplement Table S1: Distribution of AYAs with known and unknown HIV status by characteristics used in the imputation model

Characteristics	Unknown HIV status n (%)	Known HIV status n (%)
**Age [years]**		
10	162 (47.1%)	182 (52.9%)
11	152 (46.8%)	173 (53.2%)
12	160 (48.8%)	168 (51.2%)
13	190 (52.9%)	169 (47.1%)
14	210 (65%)	113 (35%)
15	210 (56.3%)	163 (43.7%)
16	233 (61.5%)	146 (38.5%)
17	259 (61.4%)	163 (38.6%)
18	276 (57.4%)	205 (42.6%)
19	284 (57.3%)	212 (42.7%)
20	337 (56.5%)	259 (43.5%)
21	401 (57.9%)	291 (42.1%)
22	451 (53.6%)	391 (46.4%)
23	530 (53.2%)	467 (46.8%)
24	576 (50.3%)	570 (49.7%)
**Sex**		
Female	2374 (53.6%)	2051 (46.4%)
Male	2057 (55.9%)	1621 (44.1%)
**Ethnicity**		
Asian	106 (68.8%)	48 (31.2%)
Black	3521 (55.3%)	2850 (44.7%)
Coloured	317 (42.7%)	426 (57.3%)
White	487 (58.3%)	348 (41.7%)
**Cancer diagnosis date**		
2004	575 (81%)	135 (19%)
2005	415 (61.8%)	257 (38.2%)
2006	492 (62.8%)	292 (37.2%)
2007	458 (57.1%)	344 (42.9%)
2008	443 (54.2%)	375 (45.8%)
2009	438 (53.3%)	383 (46.7%)
2010	369 (50%)	369 (50%)
2011	327 (43.6%)	423 (56.4%)
2012	348 (42.9%)	463 (57.1%)
2013	283 (46.2%)	329 (53.8%)
2014	283 (48.4%)	302 (51.6%)
**Cancer site**		
Anus	13 (56.5%)	10 (43.5%)
BCC	60 (85.7%)	10 (14.3%)
Bladder	18 (85.7%)	3 (14.3%)
Bone	467 (66.3%)	237 (33.7%)
Brain, CNS	192 (67.8%)	91 (32.2%)
Breast	137 (71%)	56 (29%)
Burkitt lymphoma	40 (30.5%)	91 (69.5%)

Cervix	128 (57.9%)	93 (42.1%)
Colorectal	108 (59.7%)	73 (40.3%)
Connective tissue	334 (65.1%)	179 (34.9%)
Endocrine	22 (42.3%)	30 (57.7%)
Eye	109 (69.4%)	48 (30.6%)
Gum	1 (100%)	0 (0%)
Haematology other	68 (52.7%)	61 (47.3%)
Hodgkin lymphoma	228 (39.8%)	345 (60.2%)
Ill defined	7 (100%)	0 (0%)
Kaposi Sarcoma	597 (43.9%)	764 (56.1%)
Kidney	63 (61.2%)	40 (38.8%)
Larynx	1 (25%)	3 (75%)
Leukaemia	321 (37.3%)	539 (62.7%)
Lip	8 (61.5%)	5 (38.5%)
Liver & Bile duct	70 (61.4%)	44 (38.6%)
Lung	23 (76.7%)	7 (23.3%)
Melanoma	76 (81.7%)	17 (18.3%)
Mesothelioma	3 (100%)	0 (0%)
Mouth	32 (60.4%)	21 (39.6%)
Myeloma	5 (45.5%)	6 (54.5%)
Naso-Oropharynx	95 (51.1%)	91 (48.9%)
Non Hodgkin lymphoma	253 (42.2%)	347 (57.8%)
Oesophagus	14 (66.7%)	7 (33.3%)
Other specified	42 (57.5%)	31 (42.5%)
Ovary	90 (59.6%)	61 (40.4%)
Pancreas	4 (57.1%)	3 (42.9%)
Penis	3 (50%)	3 (50%)
Placenta	53 (65.4%)	28 (34.6%)
Primary site unknown	172 (66.9%)	85 (33.1%)
Prostate	15 (83.3%)	3 (16.7%)
SCC of skin	120 (74.1%)	42 (25.9%)
Salivary gland	51 (68%)	24 (32%)
Skin other	108 (78.8%)	29 (21.2%)
Small intestine	7 (77.8%)	2 (22.2%)
Stomach	24 (58.5%)	17 (41.5%)
Testis	98 (69%)	44 (31%)
Thyroid	89 (80.2%)	22 (19.8%)
Tongue	7 (77.8%)	2 (22.2%)
Uterus	28 (57.1%)	21 (42.9%)
Vagina	10 (50%)	10 (50%)
Vulva	17 (38.6%)	27 (61.4%)

Online Supplement Table S2: Top 20 cancer in AYAs in the South African public health sector stratified by HIV status

Cancer Site	HIV positive	HIV negative	HIV unknown
NADC	697	1699	3411
ADC	1129	206	1062
Kaposi Sarcoma	786	5	617
Leukaemia	113	449	338
Bone	46	197	496
NHL	206	150	269
Hodgkin lymphoma	119	246	235
Connective tissue	54	134	348
Brain, CNS	24	75	208
Cervix	76	18	132
Breast	27	30	140
Naso-oropharynx	30	65	102
Colorectal	15	62	114
SCC of Skin	28	15	128
Eye	33	19	114
Ovary	21	43	94
Testis	7	39	102
Skin Other	11	19	114
Haematology other	15	48	73
Liver and Bile duct	10	38	76
Thyroid	7	16	96
Kidney	7	34	65

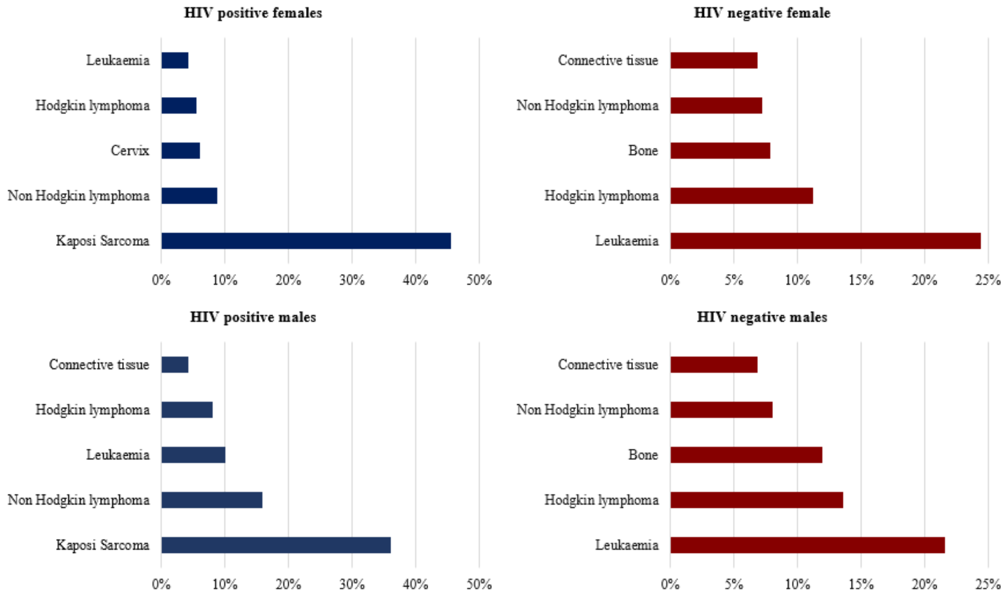

Figure S1: Top five cancers in AYAs stratified by sex and HIV status
