# Supplementary material for: Cancer in HIV-positive and HIV-negative adolescents and young adults in South Africa: a cross-sectional study
Source: BMJ Open. 2021 Oct 17;11(10):e043941. doi: 10.1136/bmjopen-2020-043941 (PMC8524277; doi:10.1136/bmjopen-2020-043941)
Supplement: Author's manuscript [file bmjopen-2020-043941.draft_revisions.pdf]

BMJ Open

BMJ Open is committed to open peer review. As part of this commitment we make the peer review history of every article we publish publicly available.

When an article is published we post the peer reviewers' comments and the authors' responses online. We also post the versions of the paper that were used during peer review. These are the versions that the peer review comments apply to.

The versions of the paper that follow are the versions that were submitted during the peer review process. They are not the versions of record or the final published versions. They should not be cited or distributed as the published version of this manuscript.

BMJ Open is an open access journal and the full, final, typeset and author-corrected version of record of the manuscript is available on our site with no access controls, subscription charges or pay-per-view fees (http://bmjopen.bmj.com).

If you have any questions on BMJ Open's open peer review process please email info.bmjopen@bmj.com

BMJ Open

Cancer in HIV-positive and HIV-negative adolescents and young adults in South Africa: a cross-sectional study

Journal:	*BMJ Open*
Manuscript ID	bmjopen-2020-043941
Article Type:	Original research
Date Submitted by the Author:	20-Aug-2020
Complete List of Authors:	Dhokotera, Tafadzwa; National Health Laboratory Service, National Cancer Registry; University of Bern Institute of Social and Preventive Medicine<br>Bohlius, Julia; University of Bern Institute of Social and Preventive Medicine<br>Egger, Matthias; University of Bern Institute of Social and Preventive Medicine; University of Cape Town School of Public Health and Family Medicine, Centre for Infectious Disease Epidemiology and Research (CIDER)<br>Spoerri, Adrian; University of Bern Institute of Social and Preventive Medicine<br>Ncayiyana, Jabulani; University of Cape Town School of Public Health and Family Medicine, Centre for Infectious Disease Epidemiology and Research (CIDER); University of the Witwatersrand School of Public Health, Division of Epidemiology and Biostatistics<br>Naidu, Gita; University of the Witwatersrand, Paediatric Haematology Oncology, Chris Hani Baragwanath Academic Hospital<br>Olago, Victor; National Health Laboratory Service, National Cancer Registry; University of the Witwatersrand School of Public Health, Division of Epidemiology and Biostatistics<br>Zwahlen , Marcel; University of Bern Institute of Social and Preventive Medicine<br>Singh, Elvira; National Health Laboratory Service, National Cancer Registry; University of the Witwatersrand School of Public Health, Division of Epidemiology and Biostatistics<br>Muchengeti, Mazvita; National Health Laboratory Service, National Cancer Registry; University of the Witwatersrand School of Public Health, Division of Epidemiology and Biostatistics
Keywords:	Epidemiology < ONCOLOGY, PUBLIC HEALTH, EPIDEMIOLOGY

SCHOLARONE™
Manuscripts

I, the Submitting Author has the right to grant and does grant on behalf of all authors of the Work (as defined in the below author licence), an exclusive licence and/or a non-exclusive licence for contributions from authors who are: i) UK Crown employees; ii) where BMJ has agreed a CC-BY licence shall apply, and/or iii) in accordance with the terms applicable for US Federal Government officers or employees acting as part of their official duties; on a worldwide, perpetual, irrevocable, royalty-free basis to BMJ Publishing Group Ltd ("BMJ") its licensees and where the relevant Journal is co-owned by BMJ to the co-owners of the Journal, to publish the Work in this journal and any other BMJ products and to exploit all rights, as set out in our licence.

The Submitting Author accepts and understands that any supply made under these terms is made by BMJ to the Submitting Author unless you are acting as an employee on behalf of your employer or a postgraduate student of an affiliated institution which is paying any applicable article publishing charge ("APC") for Open Access articles. Where the Submitting Author wishes to make the Work available on an Open Access basis (and intends to pay the relevant APC), the terms of reuse of such Open Access shall be governed by a Creative Commons licence – details of these licences and which Creative Commons licence will apply to this Work are set out in our licence referred to above.

Other than as permitted in any relevant BMJ Author's Self Archiving Policies, I confirm this Work has not been accepted for publication elsewhere, is not being considered for publication elsewhere and does not duplicate material already published. I confirm all authors consent to publication of this Work and authorise the granting of this licence.

For peer review only

Cancer in HIV-positive and HIV-negative adolescents and young adults in South Africa: a cross-sectional study

Tafadzwa Dhokotera[1,2,3], Julia Bohlius[3], Matthias Egger[3,4], Adrian Spoerri[3], Jabulani Ncayiyana[2,5], Gita Naidu[6], Victor Olago[1,2], Marcel Zwahlen,[3] Elvira Singh[1,2], Mazvita Muchengeti[1,2]

[1]National Cancer Registry, National Health Laboratory Service, Johannesburg, South Africa

[2]Division of Epidemiology and Biostatistics, School of Public Health, University of the Witwatersrand, Johannesburg, South Africa

[3]Institute of Social and Preventive Medicine (ISPM), University of Bern, Switzerland

[4]Centre for Infectious Disease Epidemiology and Research (CIDER), School of Public Health and Family Medicine, University of Cape Town, South Africa

[5]Division of Epidemiology and Biostatistics, School of Public Health and Family Medicine, University of Cape Town, South Africa

[6]Paediatric Haematology Oncology, Chris Hani Baragwanath Academic Hospital, University of the Witwatersrand, Johannesburg, South Africa

Correspondence to: Tafadzwa Dhokotera, National Cancer Registry, National Health Laboratory Service, 1 Modderfontein Road, Sandringham, Johannesburg, 2131 South Africa. Email: tafadzwad@nicd.ac.za

Key words (max 5)
Adolescents and young adults, cancer, HIV, South Africa, epidemiology

Word count: 3 314

For peer review only - http://bmjopen.bmj.com/site/about/guidelines.xhtml

Abstract (298 words (max 300))

Objective: To determine the spectrum of cancers in AYAs living with HIV in South Africa compared to their HIV negative peers.

Design: Cross sectional study with cancer data provided by the National Cancer Registry and HIV data from the National Health Laboratory Service.

Setting and participants: The NHLS is the largest provider of pathology services in the South African public sector with an estimated coverage of 80%. The NCR is a division of the NHLS. We included AYAs (aged 10-24 years) diagnosed with cancer by public health sector laboratories between 2004 and 2014 (n=8 479). We included 3 672 in the complete case analysis.

Primary and secondary outcomes: We used linked NCR and NHLS data to determine the spectrum of cancers by HIV status in AYAs. We also used multivariable logistic regression to describe the association of cancer in AYAs with HIV, adjusting for age, sex (as appropriate), ethnicity, and calendar period. Due to the large proportion of unknown HIV status we also imputed (post-hoc) the missing HIV status.

Results: From 2004-2014, 8 479 AYAs were diagnosed with cancer, HIV status was known for only 45% (n=3812); of those whose status was known, about half were HIV positive (n=1853). AYAs living with HIV were more likely to have Kaposi's sarcoma (adjusted odds ratio (aOR) 218, 95% CI 89.9-530), cervical cancer (aOR 2.18, 95% CI 1.23-3.89), non-Hodgkin's lymphoma (aOR 2.12, 95% CI 1.69-2.66), and anogenital cancers other than cervix (aOR 2.73, 95% CI 1.27-5.86). About 44% (n=1 062) of AYAs with HIV related cancers had not been tested for HIV, though they were very likely to have the disease.

Conclusions: Cancer burden in AYAs living with HIV in South Africa could be reduced by screening young women for cervical cancer and vaccinating them against human papilloma virus (HPV) infection.

For peer review only - http://bmjopen.bmj.com/site/about/guidelines.xhtml

Strength and limitations

- This is the first nationwide study in South Africa to compare the distribution of cancers in adolescents and young adults (AYAs) by HIV status.
- The record linkage and the additional results determined from the text mining process ensured that we extracted the maximum available HIV results.
- We assumed a CD4 count test indicates being HIV positive but CD4 testing maybe performed for other reasons
- Since this was a population of only AYAs diagnosed with cancer, the odds ratios could be overestimated or underestimated depending on the frequency of the cancer

For peer review only

For peer review only - http://bmjopen.bmj.com/site/about/guidelines.xhtml

INTRODUCTION

In Eastern and Southern Africa, an estimated 1.1 million adolescents aged 10-19 years are living with HIV.[1] Young people aged 15-25 years currently make up 30% of new infections.[2] Children infected with HIV perinatally are now more likely to live and to become adolescents and young adults (AYA).[3,4] The outcomes of AYAs living with HIV (AYALHIV) have been poor, mainly because it is challenging to retain them in care. They also tend to have poor virologic suppression, and their CD4 counts often drop to levels that endanger their health.[3,5–7] Co-infection with other oncogenic viruses is also common in this age group.[8,9] For those living with HIV, immunodeficiency and co-infections with other oncogenic viruses are risk factors for developing malignancies. Still, data that compare cancer risk in AYALHIV to that of their HIV negative peers in the antiretroviral therapy era is scarce in resource-limited settings.

Several studies have shown that the risk of HIV-related cancers—non-Hodgkin's lymphoma (NHL), Kaposi's sarcoma (KS) and cervical cancer (CC) is higher in AYALHIV than in HIV-negative AYA.[10–15] In the USA, the incidence of leiomyosarcoma was also higher in AYALHIV than in their peers from the general population.[10] However, most of the existing data is from settings with a low HIV burden, but we still know little about cancer burden and risk in AYALHIV in high HIV burden African countries, like South Africa.

We aimed to evaluate the spectrum and cancers associated with HIV in AYAs at a national level. The South African HIV Cancer Match (SAM) study was created to identify the risk factors and spectrum of malignancies in people living with HIV based on routine reports.[16] In this cross-sectional sub analysis, we included AYAs with a pathology-confirmed cancer diagnosis. We examined the proportion of cancer diagnoses with or without HIV infection and the risk factors for cancer in AYAs living with HIV.

For peer review only

METHODS

Study design and setting

This was a cross-sectional study with cancer data provided by the National Cancer Registry (NCR) and HIV data from the National Health Laboratory Service (NHLS). The NHLS is the largest provider of diagnostic pathology services in the South African public sector (estimated coverage is over 80% of the SA population).[17] The NHLS include the National Institute of Communicable Diseases, the National Institute of Occupational Health, and the pathology-based NCR. The Corporate Data Warehouse (CDW) is the centralised data centre of the NHLS where all the data on tests performed in its laboratories are stored.

Inclusion criteria

We included all AYAs with a primary incident cancer recorded from 2004 to 2014 in NCR records. Adolescence was defined as 10 to 19 years and young adulthood as 20 to 24 years at the time of cancer diagnosis, based on World Health Organisation (WHO) and South African Department of Health definitions[6,18]. We excluded cancer precursors and only retained laboratory-confirmed cancer records that contained the International Classification of Disease in Oncology version 3 (ICD-O-3) topography and morphology descriptions.[19] If a person had two different cancers at different sites, they were considered as two individual records (multiple primaries).

Outcome and exposure variables

The main exposure was HIV infection and the main outcome cancer diagnosis by morphological subtype. HIV status was determined from HIV diagnostic tests (ELISA, qualitative PCR and rapid HIV tests) and HIV monitoring tests (CD4 counts and HIV RNA viral loads). We assumed an individual was HIV positive if any diagnostic test was positive or if monitoring tests (CD4 cell count, viral load) were recorded. We used text mining methods to extract additional HIV results from the clinical history section of cancer pathology reports.

We used data from the CDW patient linking process which utilises probabilistic record linkage (PRL) methods to create a unique patient identifier for records belonging to the same person. As described in detail elsewhere[20], the CDW uses names, surnames and date of births as linkage variables, which are fed into the PRL linkage algorithm. First names and surnames have a weight of 40% each, and date of birth a weight of 20%. Records with a recorded national identity number are exact matches. To be considered a match, the cumulative score has to reach 90% or above. The data from the CDW has been evaluated for completeness and accuracy and validated as a good source of data for research on HIV in South Africa.[21]

For peer review only - http://bmjopen.bmj.com/site/about/guidelines.xhtml

We used NCR records to determine demographic characteristics. Where missing, the NCR imputes ethnicity based on surnames using known surname-ethnicity pairings.[22] Ethnicity was grouped into Black and Other for comparison purposes because few subjects belong to other population subgroups. We divided calendar years into three periods: the early years of combination antiretroviral therapy (ART) (2004-2008); later years (2009-2011); and, the most recent period (2012-2014). We selected cut-offs for the calendar periods to correspond with changes in ART guidelines in South Africa during the study period.[23] We grouped cancers of vulva, penis, vagina and anus as anogenital cancers other than cervical cancer. We looked at NHLs as a group, and at each of its subtypes: Burkitt lymphoma; diffuse large B cell lymphoma (DLBCL); diffuse immunoblastic large B cell lymphoma (DILBCL); follicular NHL; and, mature T cell NHL.

Data analysis

For descriptive purposes, we present sex, ethnicity (Asian, Black, Coloured and White) and age strata by HIV status (positive/negative/unknown). We show the frequency and spectrum of cancers in AYAs stratified by HIV status (positive/negative/unknown) and by sex. We used a logistic regression model to determine the association between HIV and cancer in adolescents and young adults. For each cancer, we used records without the cancer under study as the comparison group, including cancers with an infectious aetiology. We adjusted the models using age (adolescence versus (vs) young adults), sex (male vs female, except for sex-specific cancers), ethnicity (Black vs other) and ART era. We restricted our main analysis to cancers in AYAs with known HIV status, so all AYA were either HIV-positive or HIV-negative. We checked for interactions between HIV and the other factors of interest (age, sex, ethnicity and calendar period) and adjusted models for interaction analysis for age, sex, ethnicity and calendar period. To test for significance of the interaction, we used likelihood ratio tests to compare logistic regression models with and without the interaction terms at 5% significance level. Stata® 15.1 was used for all analyses (StataCorp Inc, College Station, TX, USA).

Sensitivity analysis

As a sensitivity analysis, we used multiple imputation methods to impute missing HIV results for 4431 cancer patients with unknown HIV status. We included HIV status (the primary exposure), cancer diagnosis, cancer diagnosis year, sex, age and ethnicity in our imputation model. Since ethnicity was already imputed by the NCR using surname-ethnicity pairings, we excluded records that still had missing ethnicity data (4%; n=368). We also excluded records with missing sex as they were few (0.09%; n=8). We use multivariate imputation with chained

For peer review only - http://bmjopen.bmj.com/site/about/guidelines.xhtml

equations to generate 15 imputed datasets that we combined to give a pooled estimate (odds ratio). We fit multivariable logistic regression models adjusting for age, sex, ethnicity and calendar period. We compared the results from the imputed dataset with the main complete case analysis. Table S1 in the supplement shows the distribution of known and unknown HIV status by the variables in the imputation model.

For peer review only

For peer review only - http://bmjopen.bmj.com/site/about/guidelines.xhtml

RESULTS

Over the 11 years, 8 479 AYAs were diagnosed with cancer. Over half (n=4 466) of all recorded cancer cases were YA (20-24 years), median age 20 years (interquartile range (IQR): 15-23). Girls and women made up 54% (n= 4 605) of the AYA population; most AYAs with cancer were Black 75% (n= 6 376). About 45% (n=3 819) of AYAs with cancer were assigned an HIV status; half of those with known status were HIV positive (n=1 855). When we compared AYA cancer patients with and without HIV, the median age of AYA cancer patients with HIV was 22 years (IQR: 19-23) while it was 18 years in those without HIV (IQR: 13-21). Those with HIV were more often female (67% vs 45%; p-value <0.001) and more often Black population (86% vs 64%) (Table 1). The proportion of people with unknown HIV status declined across the calendar period (Figure 1).

Table 1: Demographic characteristics of Adolescents and Young Adults with a cancer diagnosis stratified by HIV status in the South African Public Health sector, 2004-2014

	HIV negative	HIV positive	HIV unknown
	n (%)	n (%)	n (%)
**Age**			
Median age (Interquartile range) [years]	18 (13-21)	22 (19-23)	20 (16-22)
10-14	635 (32.3%)	200 (10.8%)	922 (19.8%)
15-19	585 (29.8%)	338 (18.2%)	1331 (28.6%)
20-24	744 (37.9%)	1317 (71.0%)	2407 (51.7%)
**Sex**			
Female	877 (44.7%)	1247 (67.2%)	2484 (53.3%)
Male	1087 (55.3%)	607 (32.7%)	2169 (46.5%)
Missing	0 (0%)	1 (0.1%)	7 (0.2%)
**Ethnicity**			
Asian	34 (1.7%)	14 (0.8%)	106 (2.3%)
Black	1258 (64.1%)	1593 (85.9%)	3525 (75.6%)
Coloured	323 (16.4%)	103 (5.6%)	317 (6.8%)
White	274 (14.0%)	74 (4.0%)	487 (10.5%)
Missing	75 (3.8%)	71 (3.8%)	225 (4.8%)
**Type of cancer**			
NADC	1699 (86.5%)	697 (37.6%)	3411 (73.2%)
ADC	206 (10.5%)	1129 (60.9%)	1062 (22.8%)
Primary Site unknown	59 (3.0%)	29 (1.6%)	187 (4.0%)
**ART period**			
2004-2007	594 (30.2%)	500 (27.0%)	2062 (44.2%)
2008-2012	822 (41.9%)	784 (42.3%)	1647 (35.3%)
2012-2014	548 (27.9%)	571 (30.8%)	951 (20.4%)
**Total**	1964 (100%)	1855 (100%)	4660 (100%)

NADC=non-AIDS Defining cancer. ADC=AIDS Defining Cancer. ART=Antiretroviral therapy

For peer review only - http://bmjopen.bmj.com/site/about/guidelines.xhtml

The most frequently diagnosed cancer was Kaposi sarcoma, followed by leukaemia and bone cancer (Figure2, absolute numbers in Table S2 in supplementary material). Non-AIDS Defining Cancers (NADCs) made up 68% (n= 5803) of histologically diagnosed cancers. In AYA with ADCs, 44% (n=1 062) of patients had unknown HIV status vs 59% (n=3 411) of AYA with NADC. The HIV status of 44% (n=617) of AYA diagnosed with Kaposi Sarcoma was unknown, and the HIV status of 43% (n=269) of AYA diagnosed with NHL was unknown (Figure 1). Haematological cancers were the most common cancers in AYAs without HIV: leukaemia was the most frequent diagnosis (n=449), followed by Hodgkin's lymphoma (n=246), and bone cancers (n=197). In HIV negative AYAs, the top five cancers were similar for male and female participants, but HIV-negative male AYAs had a higher proportion of Hodgkin's lymphoma and bone cancers.

Amongst those with recorded HIV status, Kaposi Sarcoma, NHL and Hodgkin's lymphoma, leukaemia, and cervical cancer were the most frequent cancers in AYAs living with HIV (Figure 1). The top five most frequent cancers amongst female AYAs with HIV were Kaposi sarcoma, NHL, cervical cancer, Hodgkin's lymphoma, and leukaemia (Figure S1 Supplementary material). For male AYAs with HIV, the most frequently diagnosed cancers were KS, NHL, leukaemia, Hodgkin's lymphoma, and connective tissue cancers. The proportion of Kaposi sarcoma cases was higher in female AYAs with HIV (71%, n=998) than in male AYAs with HIV (29%, n=409).

The logistic regression analysis revealed higher odds of AIDS Defining Cancers (ADCs) than to NADCs in AYAs with HIV (Table 2). When we compared HIV positive AYAs to HIV negative AYAs, the adjusted odds ratio for AYAs with HIV was 218 (95% CI 89.9-530) for Kaposi sarcoma, 2.18 (95% CI 1.23-3.89) for cervical cancer, and 2.12 (95% CI 1.69-2.66) for NHL. The odds of specific NHL subtypes like Burkitt lymphoma, diffuse Large B-cell lymphoma, and diffuse immunoblastic large B-cell lymphoma were higher in AYAs living with HIV than in AYAs without HIV (Table 2). Anogenital cancers other than cervical cancer were also strongly associated with HIV; adjusted OR was 2.73 (95% CI 1.27-5.86). We did not observe significant odds of leiomyosarcoma in AYAs living with HIV but, of the eight recorded leiomyosarcoma cases with a known HIV result, six were HIV positive and five were female. Odds were not higher for HIV and Hodgkin's lymphoma or HIV with liver cancer.

For peer review only - http://bmjopen.bmj.com/site/about/guidelines.xhtml

Table 2: Relationship between HIV and selected cancers amongst AYAs in the South African public health sector

Cancer site	Univariable analysis (n=3819)	Multivariable complete case analysis (n=3672)	Multivariable Imputed analysis (n=8103)
	Odds ratio (95% CI)	Odds ratio (95% CI)	Odds ratio (95% CI)
ADC	13.3 (11.2-15.8)	12.0 (9.92-14.5)	3.15 (2.71-3.65)
Kaposi Sarcoma	288 (119-696)	218 (89.9-530)	4.48 (3.64-5.52)
NHL	1.64 (1.34-2.00)	2.12 (1.69-2.66)	1.47 (1.17-1.85)
Burkitts lymphoma	1.99 (1.30-3.05)	2.65 (1.64-4.28)	2.03 (1.29-3.17
Non-Hodgkin- NOS	3.31 (1.72-6.37)	4.28 (2.06-8.92)	1.75 (1.03-2.99)
DLBCL- NOS	1.52 (1.11-2.09)	2.03 (1.42-2.90)	1.49 (1.07-2.10)
DILBCL- NOS	3.61 (1.64-7.97)	4.75 (2.01-11.3)	2.25 (1.05-4.82)
Mature T-cell- NHL	0.94 (0.36-2.44)	1.11 (0.38-3.23)	0.90 (0.36-2.24)
Follicular NHL	0.79 (0.18-3.55)	0.93 (0.12-6.89)	0.84 (0.14-5.08)
Cervical cancer	4.62 (2.75-7.75)	2.18 (1.23-3.89)	1.37 (0.92-2.04)
NADC	0.09 (0.08-0.11)	0.11 (0.09-0.13)	0.36 (0.32-0.42)
Virus-related cancers	0.56 (0.46-0.68)	0.64 (0.52-0.80)	0.80 (0.65-0.98)
Anogenital cancers other than cervix	3.91 (2.00-7.65)	2.73 (1.27-5.86)	1.59 (0.77-3.31)
Hodgkin's lymphoma	0.48 (0.38-0.60)	0.60 (0.47-0.78)	0.74 (0.58-0.94)
Liver & Bile duct	0.27 (0.14-0.55)	0.28 (0.13-0.61)	0.64 (0.33-1.24)
Virus-unrelated NADC	1.88 (1.57-2.25)	1.69 (1.38-2.07)	1.30 (1.09-1.56)
Bone	0.23 (0.16-0.32)	0.29 (0.21-0.42)	0.70 (0.51-0.93)
Brain- CNS	0.33 (0.21-0.53)	0.35 (0.20-0.60)	0.74 (0.53-1.03
Colorectal	0.25 (0.14-0.44)	0.15 (0.08-0.28)	0.51 (0.32-0.79)
Connective tissue	0.41 (0.30-0.57)	0.44 (0.31-0.64)	0.72 (0.55-0.96)
Eye	1.85 (1.05-3.27)	1.11 (0.58-2.13)	1.15 (0.69-1.91)
Haematology	0.33 (0.18-0.58)	0.63 (0.34-1.18)	0.84 (0.52-1.36)
Leukaemia	0.22 (0.18-0.27)	0.29 (0.23-0.37)	0.48 (0.39-0.61)
Leiomyosarcoma	3.18 (0.64-15.8)	2.13 (0.38-11.9)	1.24 (0.45-3.45)
Myeloma	0.79 (0.18-3.55)	0.62 (0.09-4.03)	0.64 (0.13-3.29)
Ovary	0.51 (0.30-0.87)	0.48 (0.26-0.87)	0.71 (0.45-1.12)
SCC skin	1.99 (1.06-3.74)	1.21 (0.60-2.44)	0.93 (0.56-1.56)
Skin	0.61 (0.29-1.29)	0.44 (0.19-1.02)	0.85 (0.50-1.44)
Thyroid	0.46 (0.19-1.12)	0.29 (0.11-0.77)	0.85 (0.48-1.49)
Testis	0.19 (0.08-0.42)	0.28 (0.11-0.68)	0.71 (0.39-1.31)

NHL=non-Hodgkin's Lymphoma. DLBCL= Diffuse large B-cell lymphoma. DILBCL= Diffuse immunoblastic large B-cell lymphoma. NOS= Not Otherwise Specified. The multivariable analysis is adjusted for age, sex (were applicable), ethnicity and calendar period. Imputation was done under the missing at random assumption. The variables used to impute for missing HIV status were cancer diagnosis, ethnicity, sex and cancer diagnosis year. The imputed analysis is a multivariable analysis adjusting for age, sex (were applicable), ethnicity and calendar period.

For peer review only - http://bmjopen.bmj.com/site/about/guidelines.xhtml

Interaction tests determined that age modified the odds of NHL in AYAs living with HIV; adolescents with HIV had higher odds of NHL (adjusted OR 3.17; 95% CI: 2.35-4.28) than YA with HIV (adjusted OR 1.29; 95% CI 0.93-1.79; p-value for interaction <0.0001). Ethnicity also modified the odds of Burkitt lymphoma in HIV positive AYAs; Black AYAs with HIV had higher odds of Burkitt lymphoma (adjusted OR 3.84; 95% CI: 2.10-7.04) than non-Black AYAs with HIV (adjusted OR 1.35; 95% CI: 0.43-4.28, p-value for interaction = 0.0199). In the sensitivity analysis that used the imputed dataset multivariable analysis of the imputed dataset, we observed that the, KS (adjusted OR 4.48; 95% CI: 3.64-5.52), cervical cancer (adjusted OR 1.37; 95% CI:0.92-2.04) and anogenital cancers other than cervix (adjusted OR 1.59; 95% CI: 0.77-3.31) had a substantially weaker association or no association. For all other cancers, results were similar to the main analysis of subjects with known HIV status (Table 2).

For peer review only - http://bmjopen.bmj.com/site/about/guidelines.xhtml

DISCUSSION

We observed an association amongst AYAs in the ART era between HIV and ADCs and anogenital cancers other than cervical cancer, including penile, anal, vulvar and vaginal cancers. Among those living with HIV, the proportion of KS was higher in girls and young women than in boys and young men. The combined odds of cancers not associated with HIV were higher in AYAs living with HIV than in those without HIV. We could not ascertain the HIV status of many AYAs diagnosed with HIV related cancers, however, a sensitivity analysis using imputed data yielded qualitatively similar results. We observed higher odds of Burkitt lymphoma black AYAs living with HIV compared to those without HIV and higher odds of NHL in adolescents living with HIV compared to young adults living with HIV.

The record linkage and the additional results determined from the text mining process ensured that we extracted the maximum available HIV results. Our study has several limitations. As in other HIV studies that have used CD4 counts to create HIV cohorts, we assumed that anyone who had a CD4 cell count test was HIV positive. It is possible that CD4 count tests might be performed for other reasons, but we think the risk is low since CD4 tests are usually administered after a positive HIV test. The proportion of patients whose HIV status was unknown might not be representative of the entire HIV population in South Africa, because this group includes only those who had laboratory HIV tests. Rapid test results are less likely to appear in the NHLS database (only 10% of cancers had a rapid test result). Our study shares the same limitations as the proportionate incidence analysis. Since out study population included only AYAs with cancer just like in proportionate incidence analysis, the odds ratios may have been overestimated. For the most common cancers, the odds ratio might reflect how frequently a cancer is observed and not the actual strength of association between HIV and the cancer. Using all other cancers as a comparison group may have also led to underestimating the strength of the association, especially for cancers with overlapping risk factors. However, this does not necessarily mean that the effects of the last two limitations cancel out.

It is known that the risk of ADCs is higher in AYALHIV.[10–12,14,24,25] In our study, KS was the cancer most strongly associated with HIV. HIV cohort studies have reported increased KS incidence among children and adolescents under 16.[10,12] A multicohort study found KS risk was higher in HIV positive adolescents and children from Southern Africa than in the same age group in other regions of the world.[26] In South Africa, where treatment and retention in care rates for AYAs with HIV are low[3], poorly controlled HIV infection amongst AYAs may

For peer review only - http://bmjopen.bmj.com/site/about/guidelines.xhtml

increase the odds of KS. The South African National HIV Prevalence Survey of 2017 revealed that about 60% of young adults (ages 15-24) living with HIV were not on ART.[27] Untreated AYALHIV are likely to develop immunodeficiency which increases their risk of developing KS.[26]

The risk of cervical cancer in this young adult population may be increased for several reasons. In South Africa in 2017, girls and young women were much more likely to be HIV positive (10.9% prevalence) than boys and young men (4.8%).[27] Biological factors may account for higher HIV prevalence in girls and young women, along with socio-economic factors that encourage risky sexual behaviour including transactional and intergenerational sexual relationships.[27] High prevalence and poorly controlled HIV can increase the risk of HPV co-infection in an age group less likely to be screened for cervical cancer, which in turn increases cervical cancer risk and risk of other anogenital cancers amongst AYAs. A study in the Western Cape province of South Africa found AYALHIV had higher HPV prevalence and more HPV subtypes than AYAs without HIV.[8] In contrast to other studies on cancer in AYALHIV, we observed three cervical cancer cases in AYAs between 14 and 16; two of these young women were HIV positive. HIV cohorts in South Africa and the USA have not identified cervical cancer in children and adolescents under 16,[10,14] but cervical cancer risk and incidence has been on the increase in the ART era for those between 18-24.[28] Early sexual debut, and subsequent early exposure to causative agents like HPV may explain this early presentation with cervical cancer in South Africa[8,29,30], but more studies are needed to explore this phenomenon.

Lymphomas are often misdiagnosed as tuberculosis in people living with HIV in our setting, slowing diagnosis and worsening the prognosis.[31] This might explain the significantly lower odds of Hodgkin lymphoma, a cancer associated with HIV in our study population. Like other studies, we found non-Hodgkin's lymphoma (NHL) was associated with HIV.[10,14] NHL is associated with poor adherence to ART and low rates of viral suppression, and NHL risk is high in HIV positive individuals on ART even when their disease is controlled.[14,32,33] This may be because HIV activates the CD40 receptors on B-cells like Epstein Barr virus (EBV) would in EBV related cancers such as Burkitt lymphoma.[32] We expect poor ART coverage and retention in care among AYAs with HIV increases this risk, but researchers still need to determine NHL risk in virally suppressed and non-suppressed patients in our setting. From, the interaction analysis, the odds of NHL were higher in adolescents with HIV compared to young adults with HIV. This observation could be as a result of the predominance of lymphoblastic

For peer review only - http://bmjopen.bmj.com/site/about/guidelines.xhtml

and Burkitt lymphoma, which are more common in younger ages.[34] We also found that the odds of Burkitt lymphoma were higher in HIV positive black AYAs compared to the Other ethnicities. Burkitt lymphoma in South Africa is more likely to be found in white children aged 0-14 years than in Black children[22], but this is a different age group to that in our study. We recommend physicians maintain a high suspicion index for lymphomas in AYALHIV and take care not to misdiagnose them as tuberculosis, thereby delaying care.

Other studies identified an association between leiomyosarcoma and HIVs.[13] Although not statistically significant, the odds of leiomysorcoma were higher in AYALHIV than in AYAs without HIV. Since leiomyosarcoma is rare, the association between leiomyosarcoma and HIV needs further study. Likewise, after we adjusted for the interaction of HIV with age and calendar period, AYALHIV had an increased risk of connective tissue cancer, but this finding did not reach statistical significance.

Although the proportion of subjects with unknown HIV status decreased with time, HIV testing for HIV related cancers remained low. The HIV status of many AYAs with KS, cervical cancer and NHL was unknown. In South Africa, HIV testing uptake is lower in AYAs than in adults[27] and is mostly opportunistic.[35] An AYA is most likely to be tested if they present to a health care facility with symptoms linked to a sexually transmitted infection or if a female AYA visits a reproductive health clinic.[36] Known HIV results were dependent on cancer type. The results from the complete case analysis were mostly overestimated when compared to the imputed analysis thus pointing towards differential misclassification of HIV results.

Because AYALHIV are at higher risk of ADCs and anogenital cancers and many AYAs with HIV-related cancers are not tested for HIV, HIV programmes for AYAs should extend testing coverage, link AYAs to care, and make sure to retain them. AYALHIV have a higher risk of cervical and other anogenital cancers because of the high frequency of HPV co-infection, exacerbated by sexual debut and young age. We recommend sexually active AYALHIV be screened for cervical cancer at HIV diagnosis and followed up frequently, as per the new cervical cancer guidelines so that potential CC can be identified early and treated. South Africa has already introduced an HPV vaccination programme for nine-year-old girls, and this program should be extended to AYALHIV. Those not yet infected with HPV sub-types covered by the vaccines should be vaccinated, regardless of age.

For peer review only - http://bmjopen.bmj.com/site/about/guidelines.xhtml

Conclusion

This is the first nationwide study in South Africa to compare the distribution of cancers in adolescents and young adults (AYAs) by HIV status. AIDS defining cancers (ADCs) and anogenital cancers other than cervix cancer were more common in HIV-positive than in HIV-negative AYAs. AYAs with cancer are a key population for HIV testing, however this study showed that many AYAs with ADCs are not tested for HIV. Targeted HIV testing for AYAs should be followed by the immediate start of ART after a positive diagnosis, accompanied by cervical cancer screening and vaccination against HPV to decrease cancer burden in adolescents and young adults living with HIV in South Africa.

For peer review only

Figure 1: Distribution of HIV unknowns across the study period amongst AYAs with cancer. The trend analysis for proportions was statistically significant across all strata of HIV status (p-value<0.001) for all HIV Status relative to the year of cancer diagnosis.

Figure 2: Top 20 cancer in AYAs in the South African public health sector stratified by HIV status. NADC: non-AIDS Defining Cancer. ADC: AIDS Defining Cancer. Brain, CNS: Brain Central Nervous System. SCC of skin: Squamous Cell Carcinoma of skin

For peer review only

For peer review only - http://bmjopen.bmj.com/site/about/guidelines.xhtml

REFERENCES

1 UNAIDS. Number of people livng with HIV. http://aidsinfo.unaids.org/ (accessed 8 Jun 2020).
2 WHO. WHO | HIV and youth. *WHO* Published Online First: 2017.https://www.who.int/maternal_child_adolescent/topics/adolescence/hiv/en/ (accessed 8 Jun 2020).
3 Zanoni BC, Archary M, Buchan S, *et al.* Systematic review and meta-analysis of the adolescent HIV continuum of care in South Africa: the Cresting Wave. *BMJ Glob Heal* 2016;**1**:e000004. doi:10.1136/bmjgh-2015-000004
4 UNICEF. Adolescent HIV prevention - UNICEF DATA. 2018.https://data.unicef.org/topic/hivaids/adolescents-young-people/ (accessed 24 Aug 2018).
5 Davies MA, Tsondai P, Tiffin N, *et al.* Where do HIV-infected adolescents go after transfer? – Tracking transition/transfer of HIV-infected adolescents using linkage of cohort data to a health information system platform. *J Int AIDS Soc* 2017;**20**. doi:10.7448/IAS.20.4.21668
6 Southern African HIV Clinicians Society. Guidelines for adherence to antiretroviral therapy in adolescents and young adults (expanded version): Recommendations, resources and references. Johannesburg, South Africa: 2017. http://www.sahivsoc.org/Files/Adolescent (LONG)_Guidelines_2017_WEB2.pdf (accessed 6 Apr 2018).
7 Maskew M, Bor J, Macleod W, *et al.* Adolescent HIV treatment in South Africa ' s national HIV programme : a retrospective cohort study. *Lancet HIV* 2019;**6**:e760–8. doi:10.1016/S2352-3018(19)30234-6
8 Adler DH, Wallace M, Bennie T, *et al.* Cervical dysplasia and high-risk human papillomavirus infections among HIV-infected and HIV-uninfected adolescent females in South Africa. *Infect Dis Obstet Gynecol* 2014;**2014**:498048. doi:10.1155/2014/498048
9 Kahn JA, Rudy BJ, Xu J, *et al.* Prevalence and risk factors for oral DNA tumor viruses in HIV-infected youth. *J Med Virol* 2016;**88**:1944–52. doi:10.1002/jmv.24555
10 Simard EP, Shiels MS, Bhatia K, *et al.* Long-term cancer risk among people diagnosed with AIDS during childhood. *Cancer Epidemiol Biomarkers Prev* 2012;**21**:148–54. doi:10.1158/1055-9965.EPI-11-0823
11 El-Mallawany NK, Villiera J, Kamiyango W, *et al.* Increasing Numbers of New Kaposi Sarcoma Diagnoses in HIV-Infected Children and Adolescents Despite the Wide Availability of Antiretroviral Therapy in Malawi. *Clin Infect Dis* 2017;**64**:ciw879. doi:10.1093/cid/ciw879
12 Rohner E, Schmidlin K, Zwahlen M, *et al.* Kaposi sarcoma risk in HIV-infected children and adolescents on combination antiretroviral therapy from sub-Saharan Africa, Europe, and Asia. *Clin Infect Dis* 2016;**63**:1245–53. doi:10.1093/cid/ciw519
13 Bohlius J, Foster C, Naidu G, *et al.* Cancer in adolescents and young adults living with HIV. *Curr Opin HIV AIDS* 2018;**13**:196–203. doi:10.1097/COH.0000000000000460
14 Bohlius J, Maxwell N, Spoerri A, *et al.* Incidence of AIDS-defining and other cancers in HIV-positive children in South Africa record linkage study. *Pediatr Infect Dis J* 2016;**35**:e164–70. doi:10.1097/INF.0000000000001117
15 Chiappini E, Berti E, Gianesin K, *et al.* Pediatric Human Immunodeficiency Virus infection and cancer in the Highly Active Antiretroviral Treatment (HAART) era. *Cancer Lett* 2014;**347**:38–45. doi:10.1016/j.canlet.2014.02.002
16 Sengayi M, Chen W, Spoerri A, *et al.* South African HIV cancer match study: A pilot

For peer review only - http://bmjopen.bmj.com/site/about/guidelines.xhtml

study towards precision public health. *Top Antivir Med* 2018;**26**:281s-282s.https://www.iasusa.org/sites/default/files/tam/apr_2018.pdf%0Ahttp://ovidsp.ovid.com/ovidweb.cgi?T=JS&PAGE=reference&D=emexb&NEWS=N&AN=621730054
17 Blecher M, Kollipara A, DeJager P, *et al.* Health Financing. In: Padarath A, English R, eds. *South African Health Review*. Health Systems Trust 2011. doi:10.1093/heapol/czq021
18 Child and Adolescent Health Unit. World Health Organization, Adolescent health and development. Adolesc. Heal. Dev. 2017.http://www.searo.who.int/entity/child_adolescent/topics/adolescent_health/en/ (accessed 4 Dec 2018).
19 Fritz A, Percy C, Jack A, *et al.*, editors. *International Classification of Diseases for Oncology: Third Edition*. Third. 2013. https://apps.who.int/iris/handle/10665/42344
20 Dhokotera T, Bohlius J, Spoerri A, *et al.* The burden of cancers associated with HIV in the South African public health sector, 2004-2014: a record linkage study. *Infect Agent Cancer* 2019;**14**:12. doi:10.1186/s13027-019-0228-7
21 Bassett IV, Huang M, Cloete C, *et al.* Assessing the completeness and accuracy of South African National Laboratory CD4 and viral load data: A cross-sectional study. *BMJ Open* 2018;**8**. doi:10.1136/bmjopen-2018-021506
22 Erdmann F, Kielkowski D, Schonfeld SJ, *et al.* Childhood cancer incidence patterns by race, sex and age for 2000-2006: A report from the South African National Cancer Registry. *Int J Cancer* 2015;**136**:2628–39. doi:10.1002/ijc.29308
23 Johnson LF. Access to antiretroviral treatment in South Africa 2004 - 2011. *South Afr J HIV Med* 2012;**13**:22–7. doi:10.4102/sajhivmed.v13i1.156
24 Dryden-Peterson S, Medhin H, Kebabonye-Pusoentsi M, *et al.* Cancer incidence following expansion of HIV treatment in Botswana. *PLoS One* 2015;**10**:1–13. doi:10.1371/journal.pone.0135602
25 Shiels MS, Pfeiffer RM, Engels EA. Age at cancer diagnosis among persons with aids in the United States. *Ann Intern Med* 2010;**153**:452–61. doi:10.7326/0003-4819-153-7-201010050-00008
26 Bohlius J, Valeri F, Maskew M, *et al.* Kaposi's Sarcoma in HIV-infected patients in South Africa: Multicohort study in the antiretroviral therapy era. *Int J cancer* 2014;**135**:2644–52. doi:10.1002/ijc.28894
27 Simbayi L, Zuma K, Zungu N, *et al.* South African National HIV Prevalence, Incidence and Behaviour and Communication Survey, 2017. Cape Town: 2019.
28 Rohner E, Sengayi M, Goeieman B, *et al.* Cervical cancer risk and impact of Pap-based screening in HIV-positive women on antiretroviral therapy in Johannesburg, South Africa. *Int J Cancer* 2017;**141**:488–96. doi:10.1002/ijc.30749
29 Richter L, Mabaso M, Ramjith J, *et al.* Early sexual debut: Voluntary or coerced? Evidence from longitudinal data in South Africa – the birth to twenty plus study. *South African Med J* 2015;**105**:304–7. doi:10.7196/SAMJ.8925
30 Miller CL, Nkala B, Closson K, *et al.* The Botsha Bophelo Adolescent Health Study: A profile of adolescents in Soweto, South Africa. *South Afr J HIV Med* 2017;**18**:10. doi:10.4102/sajhivmed.v18i1.731
31 Puvaneswaran B, Shoba B. Misdiagnosis of tuberculosis in patients with lymphoma. *South African Med J* 2013;**103**:32–3. doi:10.7196/SAMJ.6093
32 Epeldegui M, Vendrame E, Martínez-Maza O. HIV-associated immune dysfunction and viral infection: Role in the pathogenesis of AIDS-related lymphoma. Immunol. Res. 2010;**48**:72–83. doi:10.1007/s12026-010-8168-8
33 Achenbach CJ, Buchanan AL, Cole SR, *et al.* HIV Viremia and Incidence of Non-Hodgkin Lymphoma in Patients Successfully Treated With Antiretroviral Therapy.

For peer review only - http://bmjopen.bmj.com/site/about/guidelines.xhtml

Clin Infect Dis 2014;**58**:1599–606. doi:10.1093/cid/ciu076
34 Andrew M. Evens, DO, MS, Jane N. Winter, MD, Leo I. Gordon, MD, Brian C. H. Chiu, PhD, Richard Tsang, MD, Steven T. Rosen M. Non-Hodgkin Lymphoma. 2015.
35 Ramirez-Avila L, Nixon K, Noubary F, *et al.* Routine HIV Testing in Adolescents and Young Adults Presenting to an Outpatient Clinic in Durban, South Africa. *PLoS One* 2012;**7**:e45507. doi:10.1371/journal.pone.0045507
36 Mendelsohn AS, Gill K, Marcus R, *et al.* Sexual reproductive healthcare utilisation and HIV testing in an integrated adolescent youth centre clinic in Cape Town, South Africa. *South Afr J HIV Med* 2018;**19**. doi:10.4102/sajhivmed.v19i1.826

Authors' contributions

ME, ES, MS and JB contributed towards the study design. TD contributed towards literature search, data analysis and drafting of first version of manuscript. ES and MS contributed towards data acquisition. AS contributed towards data linkage. VO contributed towards text mining of cancer pathology reports to assign HIV status. All authors contributed towards data interpretation and critical comments on the first and subsequent drafts of the manuscript. All authors read and approved the final manuscript.

Funding statement

This work was supported by grants from the U.S. Civilian Research & Development Foundation (CRDF) Global, the National Institutes of Health administrative supplement to Existing NIH Grants and Cooperative Agreements (Parent Admin Supp) (The South African HIV Cancer Match Study; U01AI069924-09, PI Matthias Egger, co-PI Julia Bohlius) PEPFAR supplement (PI Matthias Egger), the Swiss National Science Foundation (The South African HIV cancer Match Study, 320030_169967, PI Julia Bohlius) ME was supported by special project funding (grant 17481) from the Swiss National Science Foundation. TD is supported by the European Union's Horizon 2020 research and innovation programme under the Marie Skłodowska-Curie grant agreement No 801076, through the SSPH+ Global PhD Fellowship Programme in Public Health Sciences (GlobalP3HS) of the Swiss School of Public Health

Ethics and dissemination

Permission to use the routinely collected NHLS and NCR data was sought from the relevant authorities. Ethical approval to conduct the study was granted by the University of the Witwatersrand Human Research Ethics Committee [Ethics certificate numbers (SAM: M160944) and (BCAH: M171083)].

Competing interests

The authors declare no competing interests

For peer review only - http://bmjopen.bmj.com/site/about/guidelines.xhtml

Patient and public involvement

The study is based on routinely collected laboratory data therefore no patients were involved in the design, conduct, reporting, or dissemination plans of our research. Due to the anonymous nature of the data, we cannot disseminate the results of analyses of the data directly to study participants

Patient consent for publication

Not required.

Data availability statement

The datasets used and/or analysed during the current study are available from the corresponding author upon reasonable request.

Acknowledgements

The authors would like to thank all funders, the University of the Witwatersrand, the National Health Laboratory Service (NHLS), the NHLS's Corporate Data Warehouse (special thanks to Sue Candy) and the National Cancer Registry. We would also like to thank Kali Tali for her editorial input.

For peer review only

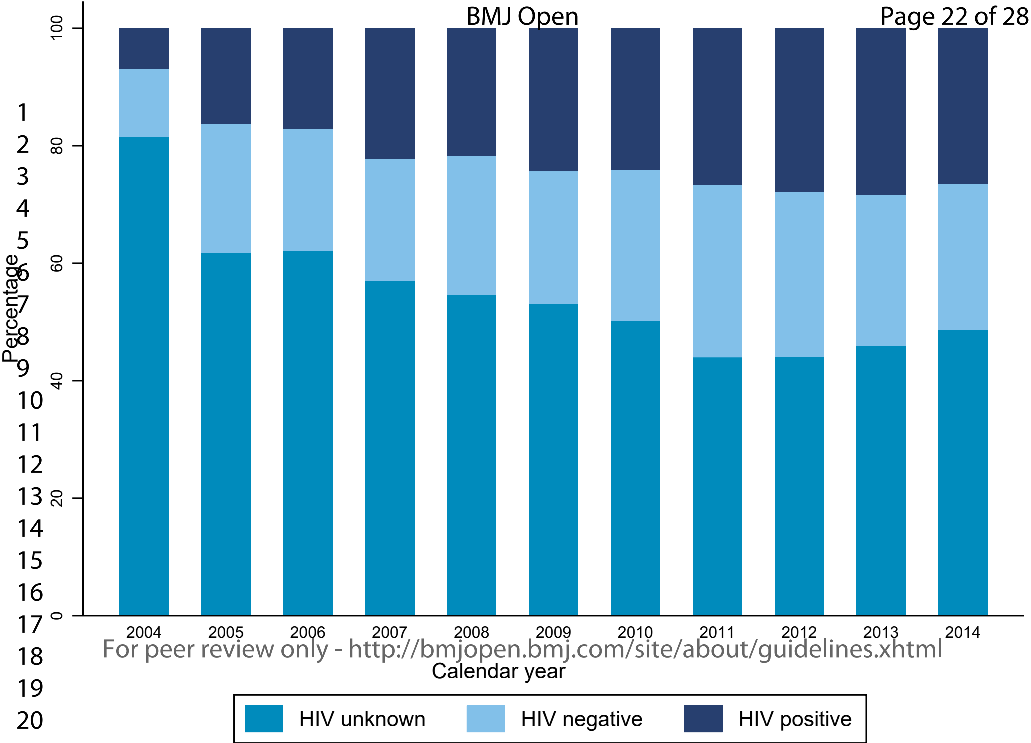

For peer review only - http://bmjopen.bmj.com/site/about/guidelines.xhtml

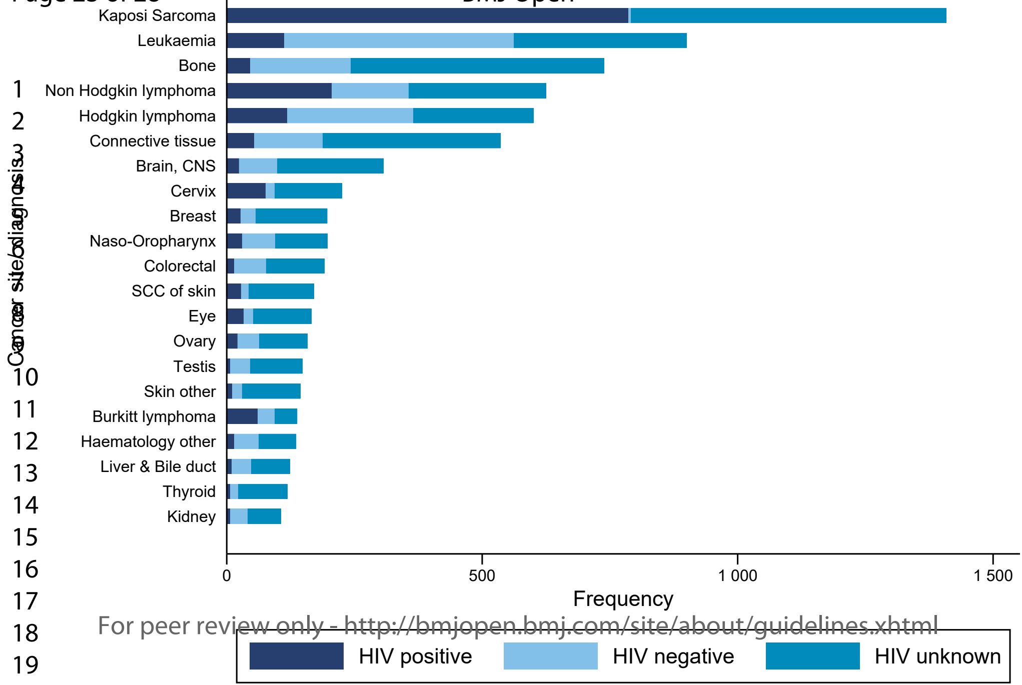

For peer review only - http://bmjopen.bmj.com/site/about/guidelines.xhtml

Online Supplement Table S1: Distribution of AYAs with known and unknown HIV status by characteristics used in the imputation model

Characteristics	Unknown HIV status n (%)	Known HIV status n (%)
**Age [years]**		
10	162 (47.1%)	182 (52.9%)
11	152 (46.8%)	173 (53.2%)
12	160 (48.8%)	168 (51.2%)
13	190 (52.9%)	169 (47.1%)
14	210 (65%)	113 (35%)
15	210 (56.3%)	163 (43.7%)
16	233 (61.5%)	146 (38.5%)
17	259 (61.4%)	163 (38.6%)
18	276 (57.4%)	205 (42.6%)
19	284 (57.3%)	212 (42.7%)
20	337 (56.5%)	259 (43.5%)
21	401 (57.9%)	291 (42.1%)
22	451 (53.6%)	391 (46.4%)
23	530 (53.2%)	467 (46.8%)
24	576 (50.3%)	570 (49.7%)
**Sex**		
Female	2374 (53.6%)	2051 (46.4%)
Male	2057 (55.9%)	1621 (44.1%)
**Ethnicity**		
Asian	106 (68.8%)	48 (31.2%)
Black	3521 (55.3%)	2850 (44.7%)
Coloured	317 (42.7%)	426 (57.3%)
White	487 (58.3%)	348 (41.7%)
**Cancer diagnosis date**		
2004	575 (81%)	135 (19%)
2005	415 (61.8%)	257 (38.2%)
2006	492 (62.8%)	292 (37.2%)
2007	458 (57.1%)	344 (42.9%)
2008	443 (54.2%)	375 (45.8%)
2009	438 (53.3%)	383 (46.7%)
2010	369 (50%)	369 (50%)
2011	327 (43.6%)	423 (56.4%)
2012	348 (42.9%)	463 (57.1%)
2013	283 (46.2%)	329 (53.8%)
2014	283 (48.4%)	302 (51.6%)
**Cancer site**		
Anus	13 (56.5%)	10 (43.5%)
BCC	60 (85.7%)	10 (14.3%)
Bladder	18 (85.7%)	3 (14.3%)
Bone	467 (66.3%)	237 (33.7%)
Brain, CNS	192 (67.8%)	91 (32.2%)
Breast	137 (71%)	56 (29%)
Burkitt lymphoma	40 (30.5%)	91 (69.5%)

For peer review only

Cervix	128 (57.9%)	93 (42.1%)
Colorectal	108 (59.7%)	73 (40.3%)
Connective tissue	334 (65.1%)	179 (34.9%)
Endocrine	22 (42.3%)	30 (57.7%)
Eye	109 (69.4%)	48 (30.6%)
Gum	1 (100%)	0 (0%)
Haematology other	68 (52.7%)	61 (47.3%)
Hodgkin lymphoma	228 (39.8%)	345 (60.2%)
Ill defined	7 (100%)	0 (0%)
Kaposi Sarcoma	597 (43.9%)	764 (56.1%)
Kidney	63 (61.2%)	40 (38.8%)
Larynx	1 (25%)	3 (75%)
Leukaemia	321 (37.3%)	539 (62.7%)
Lip	8 (61.5%)	5 (38.5%)
Liver & Bile duct	70 (61.4%)	44 (38.6%)
Lung	23 (76.7%)	7 (23.3%)
Melanoma	76 (81.7%)	17 (18.3%)
Mesothelioma	3 (100%)	0 (0%)
Mouth	32 (60.4%)	21 (39.6%)
Myeloma	5 (45.5%)	6 (54.5%)
Naso-Oropharynx	95 (51.1%)	91 (48.9%)
Non Hodgkin lymphoma	253 (42.2%)	347 (57.8%)
Oesophagus	14 (66.7%)	7 (33.3%)
Other specified	42 (57.5%)	31 (42.5%)
Ovary	90 (59.6%)	61 (40.4%)
Pancreas	4 (57.1%)	3 (42.9%)
Penis	3 (50%)	3 (50%)
Placenta	53 (65.4%)	28 (34.6%)
Primary site unknown	172 (66.9%)	85 (33.1%)
Prostate	15 (83.3%)	3 (16.7%)
SCC of skin	120 (74.1%)	42 (25.9%)
Salivary gland	51 (68%)	24 (32%)
Skin other	108 (78.8%)	29 (21.2%)
Small intestine	7 (77.8%)	2 (22.2%)
Stomach	24 (58.5%)	17 (41.5%)
Testis	98 (69%)	44 (31%)
Thyroid	89 (80.2%)	22 (19.8%)
Tongue	7 (77.8%)	2 (22.2%)
Uterus	28 (57.1%)	21 (42.9%)
Vagina	10 (50%)	10 (50%)
Vulva	17 (38.6%)	27 (61.4%)

For peer review only

Online Supplement Table S2: Top 20 cancer in AYAs in the South African public health sector stratified by HIV status

Cancer Site	HIV positive	HIV negative	HIV unknown
NADC	697	1699	3411
ADC	1129	206	1062
Kaposi Sarcoma	786	5	617
Leukaemia	113	449	338
Bone	46	197	496
NHL	206	150	269
Hodgkin lymphoma	119	246	235
Connective tissue	54	134	348
Brain, CNS	24	75	208
Cervix	76	18	132
Breast	27	30	140
Naso-oropharynx	30	65	102
Colorectal	15	62	114
SCC of Skin	28	15	128
Eye	33	19	114
Ovary	21	43	94
Testis	7	39	102
Skin Other	11	19	114
Haematology other	15	48	73
Liver and Bile duct	10	38	76
Thyroid	7	16	96
Kidney	7	34	65

For peer review only

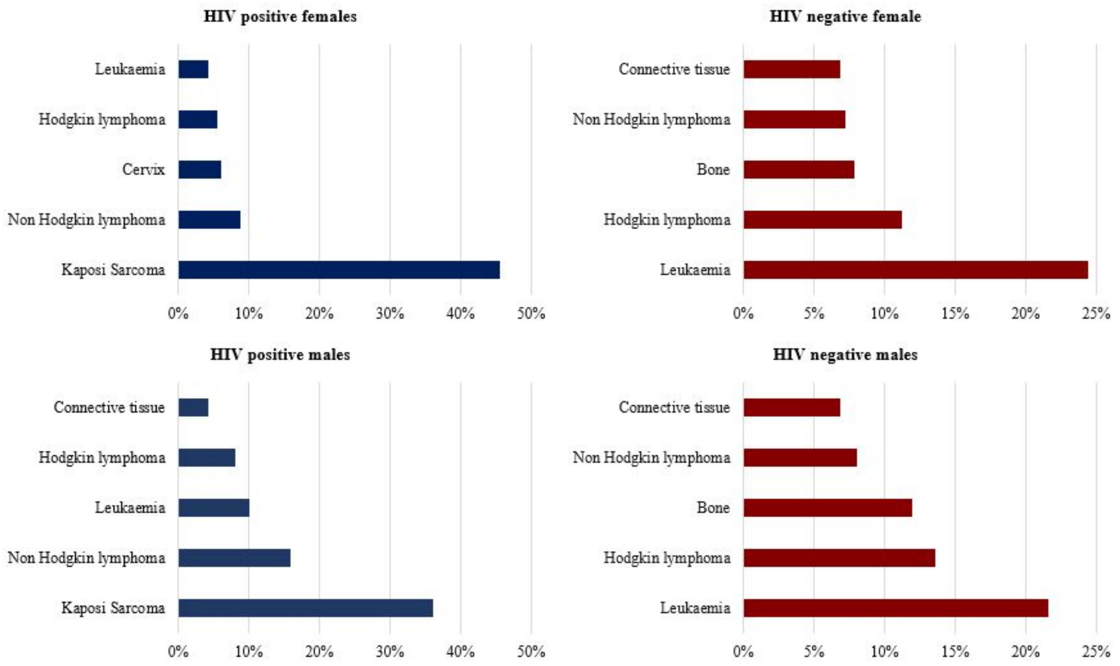

Figure S1: Top five cancers in AYAs stratified by sex and HIV status

STROBE 2007 (v4) Statement—Checklist of items that should be included in reports of *cross-sectional studies*

Section/Topic	Item #	Recommendation	Reported on page #
**Title and abstract**	1	(*a*) Indicate the study's design with a commonly used term in the title or the abstract	1-2
		(*b*) Provide in the abstract an informative and balanced summary of what was done and what was found	2
**Introduction**			
Background/rationale	2	Explain the scientific background and rationale for the investigation being reported	4
Objectives	3	State specific objectives, including any prespecified hypotheses	4
**Methods**			
Study design	4	Present key elements of study design early in the paper	5
Setting	5	Describe the setting, locations, and relevant dates, including periods of recruitment, exposure, follow-up, and data collection	5
Participants	6	(*a*) Give the eligibility criteria, and the sources and methods of selection of participants	5
Variables	7	Clearly define all outcomes, exposures, predictors, potential confounders, and effect modifiers. Give diagnostic criteria, if applicable	5
Data sources/ measurement	8*	For each variable of interest, give sources of data and details of methods of assessment (measurement). Describe comparability of assessment methods if there is more than one group	5-6
Bias	9	Describe any efforts to address potential sources of bias	6
Study size	10	Explain how the study size was arrived at	5
Quantitative variables	11	Explain how quantitative variables were handled in the analyses. If applicable, describe which groupings were chosen and why	6
Statistical methods	12	(*a*) Describe all statistical methods, including those used to control for confounding	6
		(*b*) Describe any methods used to examine subgroups and interactions	6
		(*c*) Explain how missing data were addressed	6-7
		(*d*) If applicable, describe analytical methods taking account of sampling strategy	
		(*e*) Describe any sensitivity analyses	6
**Results**			

For peer review only

Participants	13*	(a) Report numbers of individuals at each stage of study—eg numbers potentially eligible, examined for eligibility, confirmed eligible, included in the study, completing follow-up, and analysed	8
		(b) Give reasons for non-participation at each stage	
		(c) Consider use of a flow diagram	
Descriptive data	14*	(a) Give characteristics of study participants (eg demographic, clinical, social) and information on exposures and potential confounders	8
		(b) Indicate number of participants with missing data for each variable of interest	8
Outcome data	15*	Report numbers of outcome events or summary measures	8-9
Main results	16	(*a*) Give unadjusted estimates and, if applicable, confounder-adjusted estimates and their precision (eg, 95% confidence interval). Make clear which confounders were adjusted for and why they were included	10
		(*b*) Report category boundaries when continuous variables were categorized	
		(*c*) If relevant, consider translating estimates of relative risk into absolute risk for a meaningful time period	
Other analyses	17	Report other analyses done—eg analyses of subgroups and interactions, and sensitivity analyses	10-11
**Discussion**			
Key results	18	Summarise key results with reference to study objectives	12
Limitations	19	Discuss limitations of the study, taking into account sources of potential bias or imprecision. Discuss both direction and magnitude of any potential bias	12
Interpretation	20	Give a cautious overall interpretation of results considering objectives, limitations, multiplicity of analyses, results from similar studies, and other relevant evidence	12-14
Generalisability	21	Discuss the generalisability (external validity) of the study results	12
**Other information**			
Funding	22	Give the source of funding and the role of the funders for the present study and, if applicable, for the original study on which the present article is based	19

*Give information separately for cases and controls in case-control studies and, if applicable, for exposed and unexposed groups in cohort and cross-sectional studies.

Note: An Explanation and Elaboration article discusses each checklist item and gives methodological background and published examples of transparent reporting. The STROBE checklist is best used in conjunction with this article (freely available on the Web sites of PLoS Medicine at http://www.plosmedicine.org/, Annals of Internal Medicine at http://www.annals.org/, and Epidemiology at http://www.epidem.com/). Information on the STROBE Initiative is available at www.strobe-statement.org.

For peer review only

Cancer in HIV-positive and HIV-negative adolescents and young adults in South Africa: a cross-sectional study

Journal:	*BMJ Open*
Manuscript ID	bmjopen-2020-043941.R1
Article Type:	Original research
Date Submitted by the Author:	12-Jul-2021
Complete List of Authors:	Dhokotera, Tafadzwa; National Health Laboratory Service, National Cancer Registry; University of Bern Institute of Social and Preventive Medicine<br>Bohlius, Julia; University of Bern Institute of Social and Preventive Medicine<br>Egger, Matthias; University of Bern Institute of Social and Preventive Medicine; University of Cape Town School of Public Health and Family Medicine, Centre for Infectious Disease Epidemiology and Research (CIDER)<br>Spoerri, Adrian; University of Bern Institute of Social and Preventive Medicine<br>Ncayiyana, Jabulani; University of Cape Town School of Public Health and Family Medicine, Centre for Infectious Disease Epidemiology and Research (CIDER); University of the Witwatersrand School of Public Health, Division of Epidemiology and Biostatistics<br>Naidu, Gita; University of the Witwatersrand, Paediatric Haematology Oncology, Chris Hani Baragwanath Academic Hospital<br>Olago, Victor; National Health Laboratory Service, National Cancer Registry; University of the Witwatersrand School of Public Health, Division of Epidemiology and Biostatistics<br>Zwahlen , Marcel; University of Bern Institute of Social and Preventive Medicine<br>Singh, Elvira; National Health Laboratory Service, National Cancer Registry; University of the Witwatersrand School of Public Health, Division of Epidemiology and Biostatistics<br>Muchengeti, Mazvita; National Health Laboratory Service, National Cancer Registry; University of the Witwatersrand School of Public Health, Division of Epidemiology and Biostatistics
**Primary Subject Heading**:	Epidemiology
Secondary Subject Heading:	Oncology, HIV/AIDS, Public health
Keywords:	Epidemiology < ONCOLOGY, PUBLIC HEALTH, EPIDEMIOLOGY, HIV & AIDS < INFECTIOUS DISEASES

BMJ

I, the Submitting Author has the right to grant and does grant on behalf of all authors of the Work (as defined in the below author licence), an exclusive licence and/or a non-exclusive licence for contributions from authors who are: i) UK Crown employees; ii) where BMJ has agreed a CC-BY licence shall apply, and/or iii) in accordance with the terms applicable for US Federal Government officers or employees acting as part of their official duties; on a worldwide, perpetual, irrevocable, royalty-free basis to BMJ Publishing Group Ltd ("BMJ") its licensees and where the relevant Journal is co-owned by BMJ to the co-owners of the Journal, to publish the Work in this journal and any other BMJ products and to exploit all rights, as set out in our licence.

The Submitting Author accepts and understands that any supply made under these terms is made by BMJ to the Submitting Author unless you are acting as an employee on behalf of your employer or a postgraduate student of an affiliated institution which is paying any applicable article publishing charge ("APC") for Open Access articles. Where the Submitting Author wishes to make the Work available on an Open Access basis (and intends to pay the relevant APC), the terms of reuse of such Open Access shall be governed by a Creative Commons licence – details of these licences and which Creative Commons licence will apply to this Work are set out in our licence referred to above.

Other than as permitted in any relevant BMJ Author's Self Archiving Policies, I confirm this Work has not been accepted for publication elsewhere, is not being considered for publication elsewhere and does not duplicate material already published. I confirm all authors consent to publication of this Work and authorise the granting of this licence.

For peer review only

Cancer in HIV-positive and HIV-negative adolescents and young adults in South Africa: a cross-sectional study

Tafadzwa Dhokotera,[1,2,3,4,5] Julia Bohlius,[3,4,5] Matthias Egger[3,6], Adrian Spoerri,[3] Jabulani Ncayiyana,[2,7], Gita Naidu[8], Victor Olago[1,2], Marcel Zwahlen,[3] Elvira Singh[1,2], Mazvita Muchengeti[1,2,9]

[1]National Cancer Registry, National Health Laboratory Service, Johannesburg, South Africa

[2]Division of Epidemiology and Biostatistics, School of Public Health, University of the Witwatersrand, Johannesburg, South Africa

[3]Institute of Social and Preventive Medicine (ISPM), University of Bern, Bern, Switzerland

[4]Swiss Tropical and Public Health Institute, Basel, Switzerland

[5]University of Basel, Basel, Switzerland

[6]Centre for Infectious Disease Epidemiology and Research (CIDER), School of Public Health and Family Medicine, University of Cape Town, Cape Town, South Africa

[7]Division of Epidemiology and Biostatistics, School of Public Health and Family Medicine, University of Cape Town, Cape Town, South Africa

[8]Paediatric Haematology Oncology, Chris Hani Baragwanath Academic Hospital, University of the Witwatersrand, Johannesburg, South Africa

[9]South African DSI-NRF Centre of Excellence in Epidemiological Modelling and Analysis (SACEMA), Stellenbosch University, Stellenbosch, South Africa

Correspondence to: Tafadzwa Dhokotera, National Cancer Registry, National Health Laboratory Service, 1 Modderfontein Road, Sandringham, Johannesburg, 2131 South Africa. Email: tafadzwagladys.dhokotera@swisstph.ch

Key words (max 5)
Adolescents and young adults, cancer, HIV, South Africa, epidemiology

For peer review only

For peer review only - http://bmjopen.bmj.com/site/about/guidelines.xhtml

Abstract (302 words (max 300))

Objective: To determine the spectrum of cancers in adolescents and young adults (AYAs) living with and without HIV in South Africa.

Design: Cross-sectional study with cancer records provided by the National Cancer Registry (NCR) and HIV records from the National Health Laboratory Service (NHLS).

Setting and participants: The NHLS is the largest provider of pathology services in the South African public sector. The NCR is a division of the NHLS. We included AYAs (aged 10-24 years) diagnosed with cancer by public health sector laboratories between 2004 and 2014 (n=8 479). HIV status was obtained through record linkages and text mining.

Primary and secondary outcomes: We determined the spectrum of cancers by HIV status in AYAs. We used multivariable logistic regression to describe the association of cancer in AYAs with HIV, adjusting for age, sex, ethnicity, and calendar period. We imputed (post-hoc) the HIV status for AYA with unknown HIV status

Results: 8 479 AYAs were diagnosed with cancer, HIV status was known for 45% (n=3 812). Of those whose status was known, about half were HIV positive (n=1 853). AYAs living with HIV were more likely to have Kaposi's sarcoma (adjusted odds ratio (aOR) 218, 95% CI 89.9-530), cervical cancer (aOR 2.18, 95% CI 1.23-3.89), non-Hodgkin's lymphoma (aOR 2.12, 95% CI 1.69-2.66), and anogenital cancers other than cervix (aOR 2.73, 95% CI 1.27-5.86than AYAs without HIV. About 44% (n=1 062) of AYAs with HIV-related cancers had not been tested for HIV.

Conclusions: Targeted HIV testing for AYAs diagnosed with cancer, followed by immediate start of antiretroviral therapy, screening for cervical pre-cancer and vaccination against human papilloma virus is needed to decrease cancer burden in AYAs living with HIV in South Africa.

For peer review only - http://bmjopen.bmj.com/site/about/guidelines.xhtml

Strengths and limitations

- This is the first nationwide study in South Africa to compare the distribution of cancers in adolescents and young adults (AYAs) by HIV status.
- The record linkage and the additional results determined from the text mining process ensured that we extracted the maximum available HIV results.
- The record linkage and the additional results determined from the text mining process ensured that we extracted the maximum available HIV results.
- We assumed a CD4 cell count test indicates being HIV positive but CD4 testing maybe performed for other reasons.
- Since this was a population of only AYAs diagnosed with cancer, the odds ratios could be overestimated or underestimated depending on the frequency of the cancer.

For peer review only

For peer review only - http://bmjopen.bmj.com/site/about/guidelines.xhtml

INTRODUCTION

In Eastern and Southern Africa, an estimated 1.1 million adolescents aged 10-19 years are living with HIV.[1] Young people aged 15-25 years represent 30% of new infections.[2] Children infected with HIV perinatally are now more likely to live and to become adolescents and young adults (AYA) compared to the early days of the HIV epidemic.[3,4] The outcomes of AYAs living with HIV (AYALHIV) have been poor. Challenges to retain AYAs in care and lack of adherence may lead to poor virologic suppressionand low CD4 cell counts, which endangers their health.[3,5–7] Co-infection with other oncogenic viruses is also common in this age group.[8,9] For people living with HIV, immunodeficiency and co-infections with other oncogenic viruses are risk factors for developing cancer.

Several studies have shown that the risk of HIV-related cancers—non-Hodgkin's lymphoma (NHL), Kaposi's sarcoma (KS) and cervical cancer (CC) is higher in AYALHIV than in HIV negative AYA.[10–15] In the USA, the incidence of leiomyosarcoma was also higher in AYALHIV than in their peers from the general population.[10] However, most of the existing data is from settings with a low HIV burden, but we still know little about cancer burden and risk in AYALHIV in high HIV burden African countries, like South Africa. Estimating the relationship between cancer and HIV is important to determine their additional health care needs and to provide insights on potential mechanisms for prevention of cancer development in AYALHIV.

We aimed to evaluate the spectrum and cancers associated with HIV in AYAs at a national level. The South African HIV Cancer Match (SAM) study was created to identify the risk factors and spectrum of malignancies in people living with HIV based on routine reports.[16] In this cross-sectional analysis, which is a sub-project of the SAM study, we included all AYAs with a pathology-confirmed cancer diagnosis. We examined the proportion of cancer diagnoses with or without HIV infection and the factors associated with cancer in AYAs living with HIV.

For peer review only - http://bmjopen.bmj.com/site/about/guidelines.xhtml

METHODS

Study design and setting

This was a cross-sectional study with cancer data provided by the National Cancer Registry (NCR) and HIV-related laboratory data from the National Health Laboratory Service (NHLS). The NHLS is the largest provider of diagnostic pathology services in the South African public sector (estimated coverage is over 80% of the South African population).[17] The NHLS includes the National Institute of Communicable Diseases, the National Institute of Occupational Health, and the pathology-based NCR. The Corporate Data Warehouse (CDW) is the centralised data centre of the NHLS where all the data on tests performed in its laboratories are stored.

Inclusion criteria

We included all AYAs with a primary incident cancer recorded from 2004 to 2014 in NCR records irrespective of HIV status. Adolescence was defined as 10 to 19 years and young adulthood as 20 to 24 years at the time of cancer diagnosis, based on World Health Organisation (WHO) and South African Department of Health definitions.[6,18] We excluded cancer precursors and only retained laboratory-confirmed cancer records that contained the International Classification of Disease in Oncology version 3 (ICD-O-3) topography and morphology descriptions.[19] If a person had two different cancers at different sites, they were considered as two individual records (multiple primaries).

Outcome and exposure variables

The main exposure was HIV infection and the main outcome cancer diagnosis stratified by morphological type and subtype where applicable. HIV status was determined from HIV diagnostic tests (enzyme-linked immnunosorbent assay, qualitative polymerase chain reaction and rapid HIV tests) and HIV monitoring tests (CD4 cell counts and HIV RNA viral loads). We assumed an individual was HIV positive if any diagnostic test was positive or if monitoring tests (CD4 cell count, HIV RNA viral load) were recorded. We used text mining methods to extract additional HIV results from the clinical history section of cancer pathology reports as discussed in detail elsewhere.[20] We assigned the HIV status irrespective of the cancer diagnosis date.

We used deterministic and probabilistic record linkages (PRL) as well as text mining to determine HIV status. For the deterministic record linkage we used episode numbers as linkage variable. Episode number refers to tests that were requested for a patient at the same time by the health practitioner and assigned the same unique identifier. About 65% of the all linkages

For peer review only - http://bmjopen.bmj.com/site/about/guidelines.xhtml

were matched using the episode number. Using PRL, the CDW created a unique patient identifier for records belonging to the same person. As described in detail elsewhere,[21]. The CDW uses names, surnames and dates of births as linkage variables, which are fed into the PRL linkage algorithm. First names and surnames have a weight of 40% each, and date of birth a weight of 20%. Records with a recorded national identity number are exact matches. To be considered a match, the cumulative score has to reach 90% or above. The data from the CDW has been evaluated for completeness and accuracy and validated as a good source of data for research on HIV in South Africa.[22] After this, we then added the text mining data. We used NCR records to determine demographic characteristics. Where missing, the NCR imputes ethnicity based on surnames using known surname-ethnicity pairings.[23] Ethnicity was grouped into Black and Other for comparison purposes because few subjects belonged to other population subgroups. We divided calendar years into three periods: the early years of combination antiretroviral therapy (ART) (2004-2008); later years (2009-2011); and, the most recent period (2012-2014). We selected cut-offs for the calendar periods to correspond with changes in ART guidelines in South Africa during the study period.[24] We grouped cancers of vulva, penis, vagina and anus as anogenital cancers other than cervical cancer (CC). We evaluated NHL as a group, and at each of its subtypes: Burkitt lymphoma; diffuse large B cell lymphoma (DLBCL); diffuse immunoblastic large B cell lymphoma (DILBCL); follicular NHL; and, mature T cell NHL.

Data analysis

For descriptive purposes, we presented sex, ethnicity (Asian, Black, Coloured (mixed race) and White) and age stratified by HIV status (positive/negative/unknown). We then showed the frequency and spectrum of the top 20 cancers in AYAs stratified by HIV status (positive/negative/unknown) and by sex. We used a logistic regression model to determine the association between HIV and cancer in adolescents and young adults. For each cancer, we used records without the cancer under study as the comparison group, including cancers with an infectious aetiology. We adjusted the models using age (adolescence versus (vs) young adults), sex (male vs female, except for sex-specific cancers), ethnicity (Black vs other) and ART era. We restricted our main analyses to cancers in AYAs with known HIV status, so all AYA were either HIV positive or HIV negative. We assessed interactions between HIV and other factors of interest (age, sex, ethnicity and calendar period) and adjusted models for interaction analysis for age, sex, ethnicity and calendar period. To test for significance of the interaction, we used likelihood ratio tests to compare logistic regression models with and without the interaction

For peer review only - http://bmjopen.bmj.com/site/about/guidelines.xhtml

terms at 5% significance level. Stata® 15.1 was used for all analyses (StataCorp Inc, College Station, TX, USA).

Sensitivity analysis

As a sensitivity analysis, we used multiple imputation methods to impute missing HIV results for 4 431 cancer patients with unknown HIV status. We included HIV status (the primary exposure), cancer diagnosis period, sex, age and ethnicity in our imputation model. Since ethnicity was already imputed by the NCR using surname-ethnicity pairings, we excluded records that still had missing ethnicity data (4%; n=368). We also excluded records with missing sex as they were few (0.09%; n=8). We used multivariable imputation with chained equations to generate 15 imputed datasets that we combined to give a pooled estimate (odds ratio). For each cancer, we fitted multivariable logistic regression models adjusting for age, sex, ethnicity and calendar period. We compared the results from the imputed dataset with the main complete case analysis. Table S1 in the supplement compares the proportional distribution of known and unknown HIV status by the variables in the imputation model.

Patient and public involvement

The study is based on routinely collected laboratory data therefore no patients were involved in the design, conduct, reporting, or dissemination plans of our research. Due to the anonymous nature of the data, we cannot disseminate the results of analyses of the data directly to study participants.

For peer review only

For peer review only - http://bmjopen.bmj.com/site/about/guidelines.xhtml

RESULTS

Over the 11 years of study period, 8 479 AYAs were diagnosed with cancer. The median age was 20 years (interquartile range (IQR): 15-23) and over half (n=4 466) of all recorded cancer cases were diagnosed in young adults (20-24 years). Girls and women were 54% (n= 4 605) of the AYA population; most AYAs with cancer were Black 75% (n= 6 376). About 45% (n=3 819) of AYAs with cancer were assigned an HIV status; half of those with known status were HIV positive (n=1 855). When we compared AYA cancer patients with and without HIV, the median age of AYA cancer patients with HIV was 22 years (IQR: 19-23) while it was 18 years in those without HIV (IQR: 13-21). In our analysis, AYAs with HIV were more often female (67% vs 45%; p-value <0.001) and more often Black population (86% vs 64%) (Table 1) as compared to AYA without HIV. The proportion of AYA with unknown HIV status declined across the calendar periods (Figure 1).

Table 1: Demographic characteristics of Adolescents and Young Adults with a cancer diagnosis stratified by HIV status in the South African Public Health sector, 2004-2014

	HIV negative	HIV positive	HIV unknown
	n (%)	n (%)	n (%)
**Age**			
Median age (interquartile range) [years]	18 (13-21)	22 (19-23)	20 (16-22)
10-14	635 (32.3%)	200 (10.8%)	922 (19.8%)
15-19	585 (29.8%)	338 (18.2%)	1331 (28.6%)
20-24	744 (37.9%)	1317 (71.0%)	2407 (51.7%)
**Sex**			
Female	877 (44.7%)	1247 (67.2%)	2484 (53.3%)
Male	1087 (55.3%)	607 (32.7%)	2169 (46.5%)
Missing	0 (0%)	1 (0.1%)	7 (0.2%)
**Ethnicity**			
Asian	34 (1.7%)	14 (0.8%)	106 (2.3%)
Black	1258 (64.1%)	1593 (85.9%)	3525 (75.6%)
Coloured (mixed race)	323 (16.4%)	103 (5.6%)	317 (6.8%)
White	274 (14.0%)	74 (4.0%)	487 (10.5%)
Missing	75 (3.8%)	71 (3.8%)	225 (4.8%)
**Type of cancer**			
Non-AIDS defining cancer	1699 (86.5%)	697 (37.6%)	3411 (73.2%)
AIDS defining cancer	206 (10.5%)	1129 (60.9%)	1062 (22.8%)
Primary site unknown	59 (3.0%)	29 (1.6%)	187 (4.0%)
**ART calendar period**			
2004-2007	594 (30.2%)	500 (27.0%)	2062 (44.2%)
2008-2011	822 (41.9%)	784 (42.3%)	1647 (35.3%)
2012-2014	548 (27.9%)	571 (30.8%)	951 (20.4%)
**Multiple primary cancer**			
Yes	10 (0.50%)	13 (0.70%)	31 (0.67%)
No	1954 (99.5%)	1842 (99.3%)	4629 (99.3%)
**Total**	1964 (100%)	1855 (100%)	4660 (100%)

For peer review only - http://bmjopen.bmj.com/site/about/guidelines.xhtml

ART=Antiretroviral therapy. Multiple primary cancer refers to an individual with more than 1 cancer at different primary sites.

The most frequently diagnosed cancer was KS, followed by leukaemia and bone cancer (Figure 2, absolute numbers in Table S2 in supplementary material). Non-AIDS defining cancers (NADCs) made up 68% (n= 5 803) of histologically diagnosed cancers. In AYA with AIDS defining cancers (ADCs, 44% (n=1 062) of patients had unknown HIV status vs 59% (n=3 411) of AYA with NADC. The HIV status of 44% (n=617) of AYA diagnosed with KS was unknown, and the HIV status of 43% (n=269) of AYA diagnosed with NHL was unknown (Figure 1). Haematological cancers were the most common cancers in AYAs without HIV: leukaemia was the most frequent diagnosis (n=449), followed by Hodgkin's lymphoma (n=246).Bone cancers were also more common in AYAs without HIV (n=197). In HIV negative AYAs, the top five cancers were similar for male and female patients but HIV negative male AYAs had a higher proportion of Hodgkin's lymphoma and bone cancers compared to female AYAs.

Amongst those with recorded HIV status, KS, NHL and Hodgkin's lymphoma, leukaemia, and CC were the most frequent cancers in AYAs living with HIV (Figure 1). The top five most frequent cancers amongst female AYAs with HIV were KS, NHL, CC, Hodgkin's lymphoma, and leukaemia (Figure S1 Supplementary material). For male AYAs with HIV, the most frequently diagnosed cancers were KS, NHL, leukaemia, Hodgkin's lymphoma, and connective tissue cancers. The proportion of KS cases was higher in female AYAs with HIV (71%, n=998) than in male AYAs with HIV (29%, n=409).

The logistic regression analysis revealed higher odds of ADCs than to NADCs in AYAs with HIV (Table 2). When we compared HIV positive AYAs to HIV negative AYAs, the adjusted odds ratio (OR) for AYAs with HIV was 218 (95% CI 89.9-530) for KS, 2.18 (95% CI 1.23-3.89) for CC, and 2.12 (95% CI 1.69-2.66) for NHL. The odds of specific NHL subtypes like Burkitt lymphoma, DLBCL and DILBCL were higher in AYAs living with HIV than in AYAs without HIV (Table 2). Anogenital cancers other than CC were also strongly associated with HIV; adjusted OR was 2.73 (95% CI 1.27-5.86). We did not observe significant odds of leiomyosarcoma in AYAs living with HIV but, of the eight recorded leiomyosarcoma cases with a known HIV result, six were HIV positive and five were female. Odds were not high for HIV and Hodgkin's lymphoma or HIV and liver cancer.

For peer review only - http://bmjopen.bmj.com/site/about/guidelines.xhtml

Table 2: Relationship between HIV and selected cancers amongst AYAs in the South African public health sector

Cancer site	Univariable analyses (n=3819)	Multivariable complete case analyses (n=3672)	Multivariable imputed analyses (n=8103)
	Odds ratio (95% CI)	Odds ratio (95% CI)	Odds ratio (95% CI)
AIDS defining cancers	13.3 (11.2-15.8)	12.0 (9.92-14.5)	11.8 (9.75-14.3)
Kaposi's sarcoma	288 (119-696)	218 (89.9-530)	208 (83.9-519)
NHL	1.64 (1.34-2.00)	2.12 (1.69-2.66)	2.12 (1.64-2.74)
Burkitt lymphoma	1.99 (1.30-3.05)	2.65 (1.64-4.28)	2.78 (1.75-4.37)
NHL- NOS	3.31 (1.72-6.37)	4.28 (2.06-8.92)	4.00 (1.78-8.99)
DLBCL- NOS	1.52 (1.11-2.09)	2.03 (1.42-2.90)	1.97 (1.34-2.91)
DILBCL- NOS	3.61 (1.64-7.97)	4.75 (2.01-11.3)	4.80 (2.04-11.3)
Mature T-cell- NHL	0.94 (0.36-2.44)	1.11 (0.38-3.23)	1.05 (0.37-2.97)
Follicular NHL	0.79 (0.18-3.55)	0.93 (0.12-6.89)	0.96 (0.09-9.80)
Cervical cancer	4.62 (2.75-7.75)	2.18 (1.23-3.89)	2.70 (1.31-5.53)
Non-AIDS defining cancer	0.09 (0.08-0.11)	0.11 (0.09-0.13)	0.11 (0.09-0.12)
Virus-related cancers	0.56 (0.46-0.68)	0.64 (0.52-0.80)	0.61 (0.48-0.79)
Anogenital cancers other than cervix	3.91 (2.00-7.65)	2.73 (1.27-5.86)	2.61 (1.11-6.11)
Hodgkin's lymphoma	0.48 (0.38-0.60)	0.60 (0.47-0.78)	0.58 (0.44-0.78)
Liver & Bile duct	0.27 (0.14-0.55)	0.28 (0.13-0.61)	0.26 (0.11-0.64)
Virus-unrelated non AIDS defining cancers	1.88 (1.57-2.25)	1.69 (1.38-2.07)	1.70 (1.33-2.17)
Bone	0.23 (0.16-0.32)	0.29 (0.21-0.42)	0.29 (0.20-0.41)
Brain- CNS	0.33 (0.21-0.53)	0.35 (0.20-0.60)	0.37 (0.17-0.80)
Colorectal	0.25 (0.14-0.44)	0.15 (0.08-0.28)	0.15 (0.07-0.29)
Connective tissue	0.41 (0.30-0.57)	0.44 (0.31-0.64)	0.46 (0.31-0.69)
Eye	1.85 (1.05-3.27)	1.11 (0.58-2.13)	1.03 (0.45-2.32)
Haematology	0.33 (0.18-0.58)	0.63 (0.34-1.18)	0.67 (0.33-1.36)
Leukaemia	0.22 (0.18-0.27)	0.29 (0.23-0.37)	0.30 (0.23-0.39)
Leiomyosarcoma	3.18 (0.64-15.8)	2.13 (0.38-11.9)	2.01 (0.31-12.9)
Myeloma	0.79 (0.18-3.55)	0.62 (0.09-4.03)	0.63 (0.07-5.57)
Ovary	0.51 (0.30-0.87)	0.48 (0.26-0.87)	0.58 (0.31-1.08)
SCC skin	1.99 (1.06-3.74)	1.21 (0.60-2.44)	1.07 (0.50-2.27)

For peer review only - http://bmjopen.bmj.com/site/about/guidelines.xhtml

For peer review only

Skin	0.61 (0.29-1.29)	0.44 (0.19-1.02)	0.37 (0.15-0.87)
Thyroid	0.46 (0.19-1.12)	0.29 (0.11-0.77)	0.31 (0.09-1.05)
Testis	0.19 (0.08-0.42)	0.28 (0.11-0.68)	0.21 (0.07-0.57)

NHL=non-Hodgkin's lymphoma. DLBCL= Diffuse large B-cell lymphoma. DILBCL= Diffuse immunoblastic large B-cell lymphoma. NOS= Not Otherwise Specified. The multivariable analyses adjusted for age, sex (where applicable), ethnicity and calendar period. Imputation was done under the missing at random assumption for each cancer type. The variables used to impute for missing HIV status were ethnicity, sex and cancer diagnosis year. The imputed analyses are multivariable analysis adjusting for age, sex (where applicable), ethnicity and calendar period.

For peer review only

For peer review only - http://bmjopen.bmj.com/site/about/guidelines.xhtml

Interaction tests determined that age modified the odds of NHL in AYAs living with HIV; adolescents with HIV had higher odds of NHL (adjusted OR 3.17; 95% CI: 2.35-4.28) than young adults with HIV (adjusted OR 1.29; 95% CI 0.93-1.79; p-value for interaction <0.0001). Ethnicity also modified the odds of Burkitt lymphoma in HIV positive AYAs; Black AYAs with HIV had higher odds of Burkitt lymphoma (adjusted OR 3.84; 95% CI: 2.10-7.04) than non-Black AYAs with HIV (adjusted OR 1.35; 95% CI: 0.43-4.28, p-value for interaction=0.0199). In the sensitivity analysis that used the imputed dataset multivariable analysis results were similar to the main analysis of subjects with a known HIV status (Table 2). Specifically the odds of KS (adjusted OR 208; 95% CI: 83.9-519, CC (adjusted OR 2.70; 95% CI: 1.31-2.17) and anogenital cancers other than cervix (adjusted OR 2.61; 95% CI: 1.11-6.11) were higher in AYALHIV compare to HIV negative AYAs.

For peer review only - http://bmjopen.bmj.com/site/about/guidelines.xhtml

DISCUSSION

We observed an association amongst AYAs between HIV and ADCs and anogenital cancers other than CC, including penile, anal, vulvar and vaginal cancers. Among those living with HIV, the proportion of KS was higher in girls and young women than in boys and young men. The combined odds of cancers not associated with HIV were higher in AYAs living with HIV than in those without HIV. We could not ascertain the HIV status of many AYAs diagnosed with HIV-related cancers, however, a sensitivity analyses using imputed data yielded qualitatively similar results. We observed higher odds of Burkitt lymphoma Black AYAs living with HIV compared to those without HIV and higher odds of NHL in adolescents living with HIV compared to young adults living with HIV.

It is known that the risk of ADCs is higher in AYALHIV.[10–12,14,25,26] In our study, KS was the cancer most strongly associated with HIV. HIV cohort studies have reported increased KS incidence among children and adolescents under 16.[10,12] A multicohort study found KS risk was higher in HIV positive adolescents and children from Southern Africa than in the same age group in other regions of the world.[27] In South Africa, where treatment and retention in care rates for AYAs with HIV are low,[3] poorly controlled HIV infection amongst AYAs may increase the odds of KS. The South African National HIV Prevalence Survey of 2017 revealed that about 60% of young adults (ages 15-24) living with HIV were not on ART.[28] Untreated AYALHIV are likely to develop immunodeficiency which increases their risk of developing KS.[27] The higher proportion of KS in females in our study might also be a reflection of the high proportion of HIV observed in female AYAs in our study

The odds of CC and human papilloma virus (HPV) related cancers such as anogenital cancers in this young adult population may be increased for several reasons. In South Africa in 2017, girls and young women were much more likely to be HIV positive (10.9% prevalence) than boys and young men (4.8%).[28] Biological factors may account for higher HIV prevalence in girls and young women, along with socio-economic factors that encourage risky sexual behaviour including transactional and intergenerational sexual relationships.[28] High prevalence and poorly controlled HIV can increase the risk of HPV co-infection in an age group less likely to be screened for precancerous cervical lesions, which in turn increases CC risk and risk of other anogenital cancers amongst AYAs. A study in the Western Cape province of South Africa found AYALHIV had higher HPV prevalence and more high-risk HPV subtypes than AYAs without HIV.[8] In contrast to other studies on cancer in AYALHIV, we observed three CC cases

For peer review only - http://bmjopen.bmj.com/site/about/guidelines.xhtml

in AYAs aged between 14 and 16 years; two of these young women were HIV positive. HIV cohorts in South Africa and the USA have not identified CC in children and adolescents under 16 years old,[10,14] but CC risk and incidence has been on the increase in the ART era for those between 18 and 24 years old.[29] Early sexual debut, and subsequent early exposure to causative agents like HPV may explain this early presentation with CC in South Africa[8,30,31].

Lymphomas are often misdiagnosed as tuberculosis in people living with HIV in our setting, slowing lymphoma diagnosis and worsening patients' prognosis.[32] This might explain the significantly lower odds of Hodgkin lymphoma, a cancer associated with HIV in our study population. Like other studies, we found NHL was associated with HIV.[10,14] NHL is associated with poor adherence to ART and low rates of viral suppression, and NHL risk is high in HIV positive individuals on ART even when their disease is controlled.[14,33,34] This may be because HIV activates the CD40 receptors on B-cells like Epstein Barr virus (EBV) would in EBV related cancers such as Burkitt lymphoma.[33] From a national cohort study in South Africa, amongst AYAs, the incidence of NHL decreased with increasing CD4 cell counts. We expect poor ART coverage and retention in care among AYAs with HIV increases this risk, but researchers still need to determine NHL risk in virally suppressed and non-suppressed patients in our setting. From our interaction analysis, the odds of NHL were higher in adolescents with HIV compared to young adults with HIV. This observation could be as a result of the predominance of lymphoblastic and Burkitt lymphoma, which are more common in younger ages.[35] We also found that the odds of Burkitt lymphoma were higher in HIV positive black AYAs compared to the other ethnicities. Burkitt lymphoma in South Africa is more likely to be found in white children aged 0-14 years than in Black children [23], but this is a different age group to that in our study.

Other studies identified an association between leiomyosarcoma and HIVs.[13] Although not statistically significant, the odds of leiomysorcoma were higher in AYALHIV than in AYAs without HIV. Since leiomyosarcoma is rare, the association between leiomyosarcoma and HIV needs further study. Likewise, after we adjusted for the interaction of HIV with age and calendar period, AYALHIV had an increased risk of connective tissue cancer, but this finding did not reach statistical significance.

We also evaluated HIV unknowns. In South Africa, HIV testing uptake is lower in AYAs than in adults[28] and is mostly opportunistic.[36] Therefore, including this would again stress the importance of cancer patients and AYAs as a whole to be tested for HIV. Although the

For peer review only - http://bmjopen.bmj.com/site/about/guidelines.xhtml

percentage of subjects with unknown HIV status decreased over calendar periods, HIV testing for AYA diagnosed with HIV-related cancers remained low. The HIV status of many AYAs with KS, CC and NHL was unknown. An AYA is most likely to be tested if they present to a health care facility with symptoms linked to a sexually transmitted infection or if a female AYA visits a reproductive health clinic.[37].

Because AYALHIV are at higher risk of ADCs and anogenital cancers and many AYAs with HIV-related cancers are not tested for HIV, HIV programmes for AYAs should extend HIV testing coverage, link AYAs to care, and make sure to retain them. AYALHIV have a higher risk of cervical and other anogenital cancers because of the high frequency of HPV co-infection, exacerbated by sexual debut and young age. In addition we also recommend that physicians maintain a high suspicion index for lymphomas and take care no to misdiagnose them as tuberculosis, thereby delaying care.

Our study is the first nationwide study to compare the distribution of cancers in AYAs by HIV status in South Africa. The record linkage and the additional results determined from the text mining process ensured that we extracted the maximum available HIV results. Our study has several limitations. As in other HIV cohort studies[38,39] that have used CD4 cell counts to create HIV cohorts, we assumed that anyone who had a CD4 cell count test was HIV positive. It is possible, that CD4 cell count tests might be performed for other reasons. We think that this possibility is low in our study setting, because according to South African management guidelines,[40] since CD4 tests are usually administered after a positive HIV test. The proportion of patients whose HIV status was unknown might not be representative of the entire HIV population in South Africa, because our study included only those who had laboratory HIV tests. Rapid test results are less likely to appear in the NHLS database in the later years compared to the beginning of our study period (only 10% of cancers had a rapid test result). Our study shares the same limitations as the proportionate incidence analysis. Since out study population included only AYAs with cancer just like in proportionate incidence analysis, the odds ratios may have been overestimated. For the most common cancers, the odds ratio might reflect how frequently a cancer is observed and not the actual strength of association between HIV and the cancer. There is also a potential of a type one error as a result of multiple hypothesis testing on the same data set for the different cancers. However, our results are generally in line with what has been observed in adults in South and AYAs in other settings. Using all other cancers as a comparison group may have also led to underestimating the strength of the association, especially for cancers with overlapping risk factors. In addition,

For peer review only - http://bmjopen.bmj.com/site/about/guidelines.xhtml

this limits generalizability of our findings to the South African population. However, this does not necessarily mean that the effects of the last two limitations cancel out. Our study was not designed to assess associations between markers of immunosuppression and cancer risk. In our study, HIV negative individuals do not have CD4 cell count measurements and could therefore not be in included for such comparisons. Each cancer patient was assigned only one HIV-related test. Therefore, although we used CD4 cell counts to assign HIV status, we did not assess the sequence of CD4 cell counts and hence cannot establish whether the values were the baseline CD4 cell measurements or the most recent CD4 cell measurements. Lastly, those assigned HIV status using other tests would not have a CD4 cell count, which, would then result in a selection bias. Because of these reasons we did not adjust for markers of immunosuppression such as HIV RNA viral loads and CD4 cell counts in our analyses. We were also unable to assess the odds of cancer by HIV transmission route, for example vertical transmission against other transmission routes. Finally, the HPV vaccine has been rolled out in 2014 to girls between the ages of 9 and 13 years, therefore we are not able to evaluate the impact of HPV vaccination on cancer risk with our data that cover the time period 2011 to 2014.

Conclusion

This is the first nationwide study in South Africa to compare the distribution of cancers in adolescents and young adults (AYAs) by HIV status. AIDS defining cancers (ADCs) and anogenital cancers other than cervix cancer were more common in HIV positive than in HIV negative AYAs. AYAs with cancer are a key population for HIV testing, however this study suggests that many AYAs with ADCs are not tested for HIV. Targeted HIV testing for AYAs should be followed by the immediate start of ART after a positive HIV diagnosis, accompanied by screening for cervical pre-cancer and vaccination against HPV to decrease cancer burden in adolescents and young adults living with HIV in South Africa.

For peer review only

Figure 1: Distribution of HIV unknowns across the study period amongst AYAs with cancer. The trend analysis for proportions was statistically significant across all strata of HIV status (p-value<0.001) for all HIV status stratified by year of cancer diagnosis.

Figure 2: Top 21 cancer in adolescents and young adults in the South African public health sector stratified by HIV status. Brain, CNS: Brain Central Nervous System. SCC of skin: Squamous Cell Carcinoma of skin

For peer review only

REFERENCES

1. UNAIDS. Number of people livng with HIV [Internet]. [cited 2020 Jun 8];Available from: http://aidsinfo.unaids.org/
2. WHO. WHO | HIV and youth. *WHO [Internet]* 2017 [cited 2020 Jun 8];Available from: https://www.who.int/maternal_child_adolescent/topics/adolescence/hiv/en/
3. Zanoni BC, Archary M, Buchan S, Katz IT, Haberer JE. Systematic review and meta-analysis of the adolescent HIV continuum of care in South Africa: the Cresting Wave. *BMJ Glob Heal [Internet]* 2016 [cited 2018 Aug 28];1:e000004. Available from: http://gh.bmj.com/lookup/doi/10.1136/bmjgh-2015-000004
4. UNICEF. Adolescent HIV prevention - UNICEF DATA [Internet]. 2018 [cited 2018 Aug 24];Available from: https://data.unicef.org/topic/hivaids/adolescents-young-people/
5. Davies MA, Tsondai P, Tiffin N, Eley B, Rabie H, Euvrard J, Orrell C, Prozesky H, Wood R, Cogill D, Haas AD, Sohn AH, et al. Where do HIV-infected adolescents go after transfer? – Tracking transition/transfer of HIV-infected adolescents using linkage of cohort data to a health information system platform. *J Int AIDS Soc [Internet]* 2017 [cited 2018 Aug 23];20. Available from: http://doi.wiley.com/10.7448/IAS.20.4.21668
6. Southern African HIV Clinicians Society. Guidelines for adherence to antiretroviral therapy in adolescents and young adults (expanded version): Recommendations, resources and references. [Internet]. Johannesburg, South Africa: 2017 [cited 2018 Apr 6]. 9pAvailable from: http://www.sahivsoc.org/Files/Adolescent (LONG)_Guidelines_2017_WEB2.pdf
7. Maskew M, Bor J, Macleod W, Carmona S, Sherman GG, Fox MP. Adolescent HIV treatment in South Africa ' s national HIV programme : a retrospective cohort study. *Lancet HIV [Internet]* 2019;6:e760–8. Available from: http://dx.doi.org/10.1016/S2352-3018(19)30234-6
8. Adler DH, Wallace M, Bennie T, Mrubata M, Abar B, Meiring TL, Williamson AL, Bekker LG. Cervical dysplasia and high-risk human papillomavirus infections among HIV-infected and HIV-uninfected adolescent females in South Africa. *Infect Dis Obstet Gynecol [Internet]* 2014 [cited 2018 Apr 11];2014:498048. Available from: http://www.ncbi.nlm.nih.gov/pubmed/25389377
9. Kahn JA, Rudy BJ, Xu J, Kapogiannis B, Secord E, Gillison M. Prevalence and risk factors for oral DNA tumor viruses in HIV-infected youth. *J Med Virol [Internet]* 2016 [cited 2018 Aug 23];88:1944–52. Available from: http://doi.wiley.com/10.1002/jmv.24555
10. Simard EP, Shiels MS, Bhatia K, Engels EA. Long-term cancer risk among people diagnosed with AIDS during childhood. *Cancer Epidemiol Biomarkers Prev [Internet]* 2012 [cited 2018 Aug 24];21:148–54. Available from: http://www.ncbi.nlm.nih.gov/pubmed/22068287
11. El-Mallawany NK, Villiera J, Kamiyango W, Mhango J, Slone JS, Mehta PS, Kazembe PN, Scheurer ME. Increasing Numbers of New Kaposi Sarcoma Diagnoses in HIV-Infected Children and Adolescents Despite the Wide Availability of Antiretroviral Therapy in Malawi. *Clin Infect Dis [Internet]* 2017 [cited 2018 Jun 21];64:ciw879. Available from: https://academic.oup.com/cid/article-lookup/doi/10.1093/cid/ciw879
12. Rohner E, Schmidlin K, Zwahlen M, Chakraborty R, Clifford G, Obel N, Grabar S, Verbon A, Noguera-Julian A, Judd A, Collins IJ, Rojo P, et al. Kaposi sarcoma risk in HIV-infected children and adolescents on combination antiretroviral therapy from sub-Saharan Africa, Europe, and Asia. *Clin Infect Dis [Internet]* 2016 [cited 2018 Jun

For peer review only - http://bmjopen.bmj.com/site/about/guidelines.xhtml

21];63:1245–53. Available from: http://www.ncbi.nlm.nih.gov/pubmed/27578823

13. Bohlius J, Foster C, Naidu G, Sengayi M, Turkova A. Cancer in adolescents and young adults living with HIV. *Curr Opin HIV AIDS* 2018;13:196–203.
14. Bohlius J, Maxwell N, Spoerri A, Wainwright R, Sawry S, Poole J, Eley B, Prozesky H, Rabie H, Garone D, Technau K-GG, Maskew M, et al. Incidence of AIDS-defining and other cancers in HIV-positive children in South Africa record linkage study. *Pediatr Infect Dis J [Internet]* 2016 [cited 2018 Mar 19];35:e164–70. Available from: http://insights.ovid.com/crossref?an=00006454-201606000-00012
15. Chiappini E, Berti E, Gianesin K, Petrara MR, Galli L, Giaquinto C, de Martino M, De Rossi A. Pediatric Human Immunodeficiency Virus infection and cancer in the Highly Active Antiretroviral Treatment (HAART) era. *Cancer Lett [Internet]* 2014 [cited 2017 Oct 16];347:38–45. Available from: http://www.sciencedirect.com/science/article/pii/S0304383514000937
16. Sengayi M, Chen W, Spoerri A, Singh E, Egger M. South African HIV cancer match study: A pilot study towards precision public health. *Top Antivir Med [Internet]* 2018;26:281s-282s. Available from: https://www.iasusa.org/sites/default/files/tam/apr_2018.pdf%0Ahttp://ovidsp.ovid.com/ovidweb.cgi?T=JS&PAGE=reference&D=emexb&NEWS=N&AN=621730054
17. Blecher M, Kollipara A, DeJager P, Zulu N. Health Financing [Internet]. In: Padarath A, English R, eds. South African Health Review. Health Systems Trust, 2011. Available from: http://search.ebscohost.com/login.aspx?direct=true&db=lah&AN=20053168600&site=ehost-live%0Ahttp://www.hst.org.za/generic/29%0Ahttps://academic.oup.com/heapol/article-lookup/doi/10.1093/heapol/czq021
18. Child and Adolescent Health Unit. World Health Organization, Adolescent health and development [Internet]. *Adolesc. Heal. Dev.*2017 [cited 2018 Dec 4];Available from: http://www.searo.who.int/entity/child_adolescent/topics/adolescent_health/en/
19. Fritz A, Percy C, Jack A, Shanmugaratnam K, Sobin L, Parkin DM, Whelan S, eds. International Classification of Diseases for Oncology: Third Edition [Internet]. Third. 2013. 1–240pAvailable from: https://apps.who.int/iris/handle/10665/42344
20. Dhokotera T, Bohlius J, Spoerri A, Egger M, Ncayiyana J, Olago V, Singh E, Sengayi M. The burden of cancers associated with HIV in the South African public health sector, 2004-2014: A record linkage study [Internet]. *Infect. Agent. Cancer*2019 [cited 2021 Mar 3];14:12. Available from: https://infectagentscancer.biomedcentral.com/articles/10.1186/s13027-019-0228-7
21. Dhokotera T, Bohlius J, Spoerri A, Egger M, Ncayiyana J, Olago V, Singh E, Sengayi M. The burden of cancers associated with HIV in the South African public health sector, 2004-2014: a record linkage study. *Infect Agent Cancer [Internet]* 2019;14:12. Available from: https://infectagentscancer.biomedcentral.com/articles/10.1186/s13027-019-0228-7
22. Bassett IV, Huang M, Cloete C, Candy S, Giddy J, Frank SC, Parker RA. Assessing the completeness and accuracy of South African National Laboratory CD4 and viral load data: A cross-sectional study. *BMJ Open* 2018;8.
23. Erdmann F, Kielkowski D, Schonfeld SJ, Kellett P, Stanulla M, Dickens C, Kaatsch P, Singh E, Schüz J. Childhood cancer incidence patterns by race, sex and age for 2000-2006: A report from the South African National Cancer Registry. *Int J Cancer [Internet]* 2015 [cited 2020 Mar 24];136:2628–39. Available from: http://doi.wiley.com/10.1002/ijc.29308
24. Johnson LF. Access to antiretroviral treatment in South Africa 2004 - 2011. *South Afr*

For peer review only - http://bmjopen.bmj.com/site/about/guidelines.xhtml

J HIV Med [Internet] 2012 [cited 2019 Apr 17];13:22–7. Available from: https://sajhivmed.org.za/index.php/hivmed/article/view/156/261

25. Dryden-Peterson S, Medhin H, Kebabonye-Pusoentsi M, Seage GR, Suneja G, Kayembe MKA, Mmalane M, Rebbeck T, Rider JR, Essex M, Lockman S. Cancer incidence following expansion of HIV treatment in Botswana. *PLoS One* 2015;10:1–13.
26. Shiels MS, Pfeiffer RM, Engels EA. Age at cancer diagnosis among persons with aids in the United States. *Ann Intern Med [Internet]* 2010 [cited 2018 Aug 31];153:452–61. Available from: http://annals.org/article.aspx?doi=10.7326/0003-4819-153-7-201010050-00008
27. Bohlius J, Valeri F, Maskew M, Prozesky H, Garone D, Sengayi M, Fox MP, Davies M-AA, Egger M. Kaposi's Sarcoma in HIV-infected patients in South Africa: Multicohort study in the antiretroviral therapy era. *Int J cancer [Internet]* 2014 [cited 2018 Apr 6];135:2644–52. Available from: https://onlinelibrary.wiley.com/doi/pdf/10.1002/ijc.28894
28. Simbayi LC, Zuma K, Zungu N, Moyo S, Marinda E, Jooste S, Mabaso M, Ramlagan S, North A, Van Zyl J, Mohlabane N, Dietrich C, et al. South African National HIV Prevalence, Incidence, Behaviour and Communication Survey, 2017 [Internet]. Cape Town: 2019. Available from: http://www.hsrc.ac.za/en/departments/hsc/National_HIV_Survey
29. Rohner E, Sengayi M, Goeieman B, Michelow P, Firnhaber C, Maskew M, Bohlius J. Cervical cancer risk and impact of Pap-based screening in HIV-positive women on antiretroviral therapy in Johannesburg, South Africa. *Int J Cancer* 2017;141:488–96.
30. Richter L, Mabaso M, Ramjith J, Norris SA. Early sexual debut: Voluntary or coerced? Evidence from longitudinal data in South Africa – the birth to twenty plus study. *South African Med J [Internet]* 2015 [cited 2018 Sep 19];105:304–7. Available from: http://www.samj.org.za/index.php/samj/article/view/8925
31. Miller CL, Nkala B, Closson K, Chia J, Cui Z, Palmer A, Hogg R, Kaida A, Gray G, Dietrich J. The Botsha Bophelo Adolescent Health Study: A profile of adolescents in Soweto, South Africa. *South Afr J HIV Med [Internet]* 2017 [cited 2018 Sep 19];18:10. Available from: https://sajhivmed.org.za/index.php/hivmed/article/view/731/1018
32. Puvaneswaran B, Shoba B. Misdiagnosis of tuberculosis in patients with lymphoma. *South African Med J* 2013;103:32–3.
33. Epeldegui M, Vendrame E, Martínez-Maza O. HIV-associated immune dysfunction and viral infection: Role in the pathogenesis of AIDS-related lymphoma [Internet]. *Immunol. Res.*2010 [cited 2018 Sep 19];48:72–83. Available from: http://www.ncbi.nlm.nih.gov/pubmed/20717742
34. Achenbach CJ, Buchanan AL, Cole SR, Hou L, Mugavero MJ, Crane HM, Moore RD, Haubrich RH, Gopal S, Eron JJ, Hunt PW, Rodriguez B, et al. HIV Viremia and Incidence of Non-Hodgkin Lymphoma in Patients Successfully Treated With Antiretroviral Therapy. *Clin Infect Dis [Internet]* 2014 [cited 2018 Sep 14];58:1599–606. Available from: https://academic.oup.com/cid/article/58/11/1599/328575
35. Andrew M. Evens, DO, MS, Jane N. Winter, MD, Leo I. Gordon, MD, Brian C. H. Chiu, PhD, Richard Tsang, MD, Steven T. Rosen M. Non-Hodgkin Lymphoma. 2015;
36. Ramirez-Avila L, Nixon K, Noubary F, Giddy J, Losina E, Walensky RP, Bassett I V. Routine HIV Testing in Adolescents and Young Adults Presenting to an Outpatient Clinic in Durban, South Africa. *PLoS One [Internet]* 2012 [cited 2019 Aug 2];7:e45507. Available from: https://dx.plos.org/10.1371/journal.pone.0045507
37. Mendelsohn AS, Gill K, Marcus R, Robbertze D, Van de Venter C, Mendel E, Mzukwa L, Bekker L-G. Sexual reproductive healthcare utilisation and HIV testing in

For peer review only - http://bmjopen.bmj.com/site/about/guidelines.xhtml

an integrated adolescent youth centre clinic in Cape Town, South Africa. *South Afr J HIV Med* 2018;19.

38. Bassett I V., Huang M, Cloete C, Candy S, Giddy J, Frank SC, Freedberg KA, Losina E, Walensky RP, Parker RA. Using national laboratory data to assess cumulative frequency of linkage after transfer to community-based HIV clinics in South Africa. *J Int AIDS Soc [Internet]* 2019 [cited 2020 Aug 12];22. Available from: https://pubmed.ncbi.nlm.nih.gov/31243898/
39. Bor J, MacLeod W, Oleinik K, Potter J, Brennan A, Candy S, Maskew M, Fox MP, Sanne I, Stevens WS, Carmona S. Building a National HIV Cohort from Routine Laboratory Data: Probabilistic Record-Linkage with Graphs. *bioRxiv [Internet]* 2018 [cited 2019 Jan 17];450304. Available from: https://www.biorxiv.org/content/early/2018/11/02/450304.abstract
40. South African National Department of Health. Clinical guidelines for the management of HIV and AIDS in adults and adolescents [Internet]. 2010 [cited 2018 Oct 5]. 42pAvailable from: http://www.sahivsoc.org/Files/Clinical_Guidelines_for_the_Management_of_HIV_AIDS_in_Adults_Adolescents_2010.pdf

For peer review only

Authors' contributions

ME, ES, MM and JB contributed towards the study design. TD contributed towards literature search, data analysis and drafting of first version of manuscript. ES and MM contributed towards data acquisition. AS contributed towards data linkage. VO contributed towards text mining of cancer pathology reports to assign HIV status. MZ contributed towards data analysis. JN, GN and all authors contributed towards data interpretation and critical comments on the first and subsequent drafts of the manuscript. All authors read and approved the final manuscript.

Funding statement

This work was supported by grants from the U.S. Civilian Research & Development Foundation (CRDF) Global, the National Institutes of Health administrative supplement to Existing NIH Grants and Cooperative Agreements (Parent Admin Supp) (The South African HIV Cancer Match Study; U01AI069924-09, PI Matthias Egger, co-PI Julia Bohlius) PEPFAR supplement (PI Matthias Egger), the Swiss National Science Foundation (The South African HIV cancer Match Study, 320030_169967, PI Julia Bohlius) ME was supported by special project funding (grant 17481) from the Swiss National Science Foundation. TD is supported by the European Union's Horizon 2020 research and innovation programme under the Marie Skłodowska-Curie grant agreement No 801076, through the SSPH+ Global PhD Fellowship Programme in Public Health Sciences (GlobalP3HS) of the Swiss School of Public Health.

Ethics and dissemination

For peer review only - http://bmjopen.bmj.com/site/about/guidelines.xhtml

Permission to use the routinely collected NHLS and NCR data was sought from the relevant authorities. Ethical approval to conduct the study was granted by the University of the Witwatersrand Human Research Ethics Committee [Ethics certificate numbers (SAM: M160944) and (BCAH: M171083)].

Competing interests

The authors declare no competing interests

Patient consent for publication

Not required.

Data availability statement

The datasets used and/or analysed during the current study are available from the corresponding author upon reasonable request.

Acknowledgements

The authors would like to thank all funders, the University of the Witwatersrand, the National Health Laboratory Service (NHLS), the NHLS's Corporate Data Warehouse (special thanks to Sue Candy) and the National Cancer Registry. We would also like to thank Kali Tali for her editorial input.

For peer review only

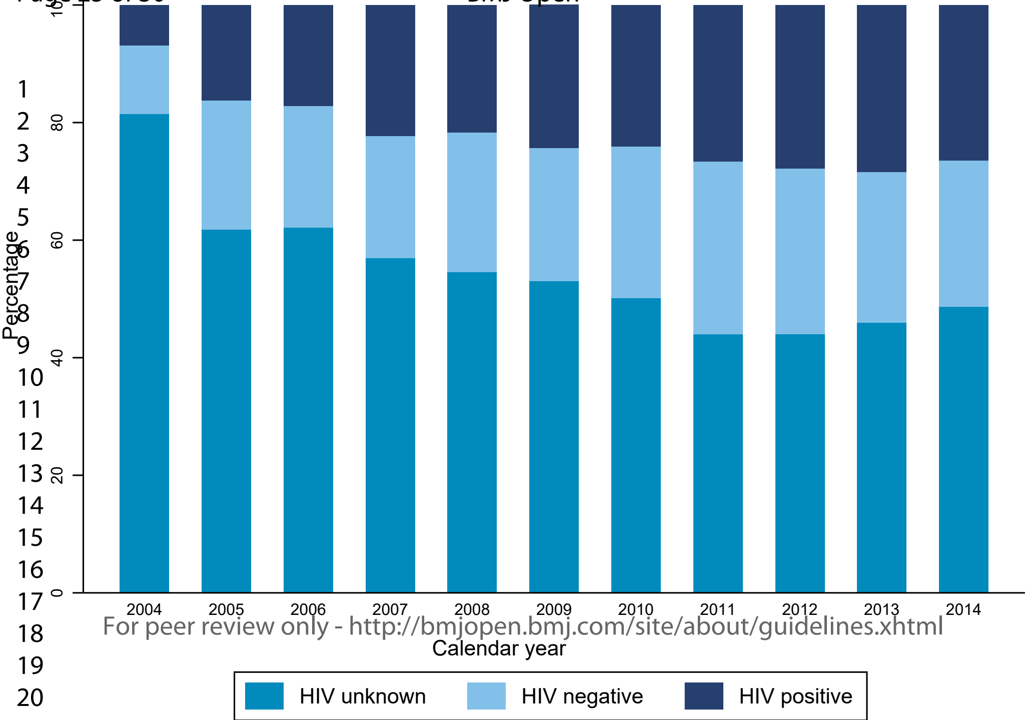

For peer review only - http://bmjopen.bmj.com/site/about/guidelines.xhtml

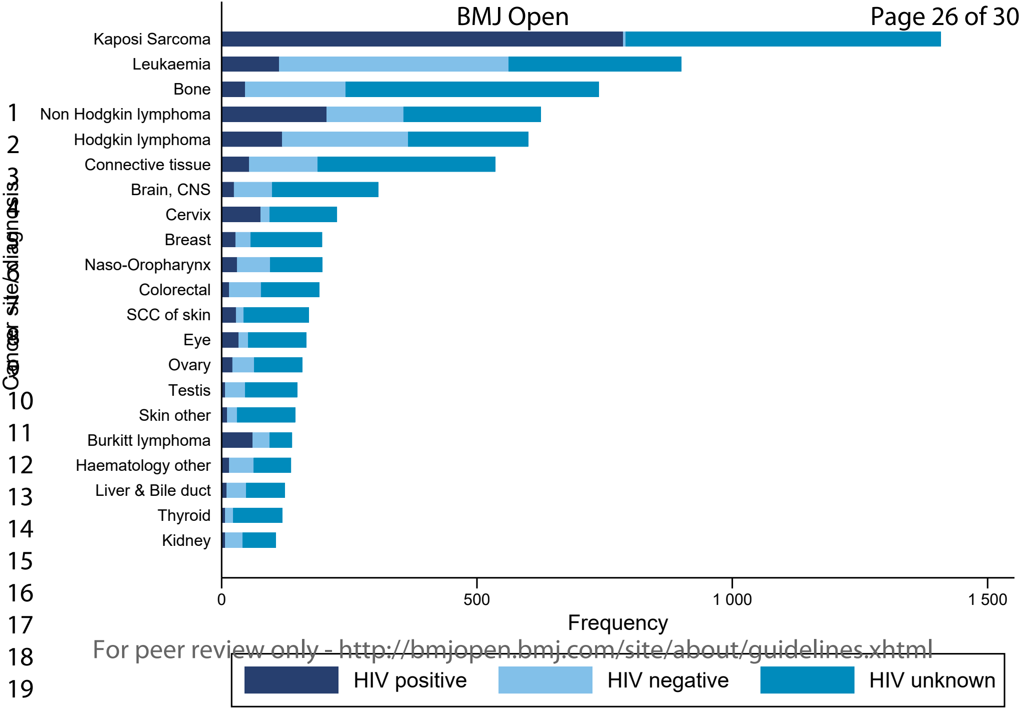

For peer review only - http://bmjopen.bmj.com/site/about/guidelines.xhtml

Online Supplement Table S1: Distribution of AYAs with known and unknown HIV status by characteristics used in the imputation model

Characteristics	Unknown HIV status n (%)	Known HIV status n (%)
**Age [years]**		
10	162 (47.1%)	182 (52.9%)
11	152 (46.8%)	173 (53.2%)
12	160 (48.8%)	168 (51.2%)
13	190 (52.9%)	169 (47.1%)
14	210 (65%)	113 (35%)
15	210 (56.3%)	163 (43.7%)
16	233 (61.5%)	146 (38.5%)
17	259 (61.4%)	163 (38.6%)
18	276 (57.4%)	205 (42.6%)
19	284 (57.3%)	212 (42.7%)
20	337 (56.5%)	259 (43.5%)
21	401 (57.9%)	291 (42.1%)
22	451 (53.6%)	391 (46.4%)
23	530 (53.2%)	467 (46.8%)
24	576 (50.3%)	570 (49.7%)
**Sex**		
Female	2374 (53.6%)	2051 (46.4%)
Male	2057 (55.9%)	1621 (44.1%)
**Ethnicity**		
Asian	106 (68.8%)	48 (31.2%)
Black	3521 (55.3%)	2850 (44.7%)
Coloured	317 (42.7%)	426 (57.3%)
White	487 (58.3%)	348 (41.7%)
**Cancer diagnosis date**		
2004	575 (81%)	135 (19%)
2005	415 (61.8%)	257 (38.2%)
2006	492 (62.8%)	292 (37.2%)
2007	458 (57.1%)	344 (42.9%)
2008	443 (54.2%)	375 (45.8%)
2009	438 (53.3%)	383 (46.7%)
2010	369 (50%)	369 (50%)
2011	327 (43.6%)	423 (56.4%)
2012	348 (42.9%)	463 (57.1%)
2013	283 (46.2%)	329 (53.8%)
2014	283 (48.4%)	302 (51.6%)
**Cancer site**		
Anus	13 (56.5%)	10 (43.5%)
BCC	60 (85.7%)	10 (14.3%)
Bladder	18 (85.7%)	3 (14.3%)
Bone	467 (66.3%)	237 (33.7%)
Brain, CNS	192 (67.8%)	91 (32.2%)
Breast	137 (71%)	56 (29%)
Burkitt lymphoma	40 (30.5%)	91 (69.5%)

For peer review only

Cervix	128 (57.9%)	93 (42.1%)
Colorectal	108 (59.7%)	73 (40.3%)
Connective tissue	334 (65.1%)	179 (34.9%)
Endocrine	22 (42.3%)	30 (57.7%)
Eye	109 (69.4%)	48 (30.6%)
Gum	1 (100%)	0 (0%)
Haematology other	68 (52.7%)	61 (47.3%)
Hodgkin lymphoma	228 (39.8%)	345 (60.2%)
Ill defined	7 (100%)	0 (0%)
Kaposi Sarcoma	597 (43.9%)	764 (56.1%)
Kidney	63 (61.2%)	40 (38.8%)
Larynx	1 (25%)	3 (75%)
Leukaemia	321 (37.3%)	539 (62.7%)
Lip	8 (61.5%)	5 (38.5%)
Liver & Bile duct	70 (61.4%)	44 (38.6%)
Lung	23 (76.7%)	7 (23.3%)
Melanoma	76 (81.7%)	17 (18.3%)
Mesothelioma	3 (100%)	0 (0%)
Mouth	32 (60.4%)	21 (39.6%)
Myeloma	5 (45.5%)	6 (54.5%)
Naso-Oropharynx	95 (51.1%)	91 (48.9%)
Non Hodgkin lymphoma	253 (42.2%)	347 (57.8%)
Oesophagus	14 (66.7%)	7 (33.3%)
Other specified	42 (57.5%)	31 (42.5%)
Ovary	90 (59.6%)	61 (40.4%)
Pancreas	4 (57.1%)	3 (42.9%)
Penis	3 (50%)	3 (50%)
Placenta	53 (65.4%)	28 (34.6%)
Primary site unknown	172 (66.9%)	85 (33.1%)
Prostate	15 (83.3%)	3 (16.7%)
SCC of skin	120 (74.1%)	42 (25.9%)
Salivary gland	51 (68%)	24 (32%)
Skin other	108 (78.8%)	29 (21.2%)
Small intestine	7 (77.8%)	2 (22.2%)
Stomach	24 (58.5%)	17 (41.5%)
Testis	98 (69%)	44 (31%)
Thyroid	89 (80.2%)	22 (19.8%)
Tongue	7 (77.8%)	2 (22.2%)
Uterus	28 (57.1%)	21 (42.9%)
Vagina	10 (50%)	10 (50%)
Vulva	17 (38.6%)	27 (61.4%)

For peer review only

Online Supplement Table S2: Top 20 cancer in AYAs in the South African public health sector stratified by HIV status

Cancer Site	HIV positive	HIV negative	HIV unknown
NADC	697	1699	3411
ADC	1129	206	1062
Kaposi Sarcoma	786	5	617
Leukaemia	113	449	338
Bone	46	197	496
NHL	206	150	269
Hodgkin lymphoma	119	246	235
Connective tissue	54	134	348
Brain, CNS	24	75	208
Cervix	76	18	132
Breast	27	30	140
Naso-oropharynx	30	65	102
Colorectal	15	62	114
SCC of Skin	28	15	128
Eye	33	19	114
Ovary	21	43	94
Testis	7	39	102
Skin Other	11	19	114
Haematology other	15	48	73
Liver and Bile duct	10	38	76
Thyroid	7	16	96
Kidney	7	34	65

For peer review only

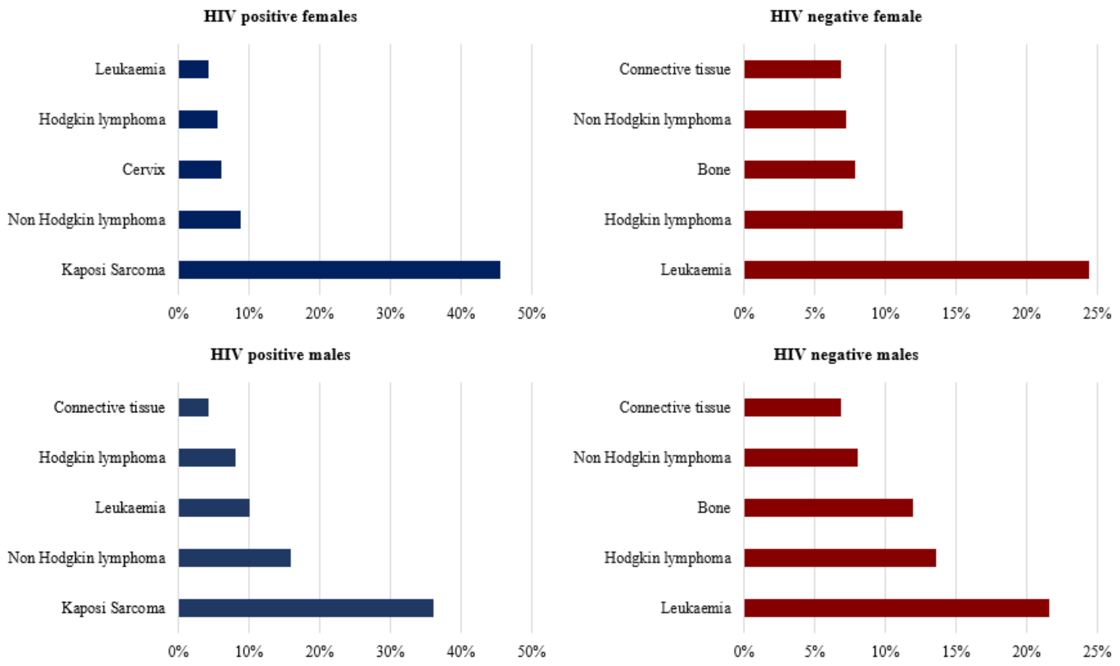

Figure S1: Top five cancers in AYAs stratified by sex and HIV status

only

STROBE 2007 (v4) Statement—Checklist of items that should be included in reports of *cross-sectional studies*

Section/Topic	Item #	Recommendation	Reported on page #
**Title and abstract**	1	(*a*) Indicate the study's design with a commonly used term in the title or the abstract	1-2
		(*b*) Provide in the abstract an informative and balanced summary of what was done and what was found	2
**Introduction**			
Background/rationale	2	Explain the scientific background and rationale for the investigation being reported	4
Objectives	3	State specific objectives, including any prespecified hypotheses	4
**Methods**			
Study design	4	Present key elements of study design early in the paper	5
Setting	5	Describe the setting, locations, and relevant dates, including periods of recruitment, exposure, follow-up, and data collection	5
Participants	6	(*a*) Give the eligibility criteria, and the sources and methods of selection of participants	5
Variables	7	Clearly define all outcomes, exposures, predictors, potential confounders, and effect modifiers. Give diagnostic criteria, if applicable	5
Data sources/ measurement	8*	For each variable of interest, give sources of data and details of methods of assessment (measurement). Describe comparability of assessment methods if there is more than one group	5-6
Bias	9	Describe any efforts to address potential sources of bias	6
Study size	10	Explain how the study size was arrived at	5
Quantitative variables	11	Explain how quantitative variables were handled in the analyses. If applicable, describe which groupings were chosen and why	6
Statistical methods	12	(*a*) Describe all statistical methods, including those used to control for confounding	6
		(*b*) Describe any methods used to examine subgroups and interactions	6
		(*c*) Explain how missing data were addressed	6-7
		(*d*) If applicable, describe analytical methods taking account of sampling strategy	
		(*e*) Describe any sensitivity analyses	6
**Results**			

For peer review only

Participants	13*	(a) Report numbers of individuals at each stage of study—eg numbers potentially eligible, examined for eligibility, confirmed eligible, included in the study, completing follow-up, and analysed	8
		(b) Give reasons for non-participation at each stage	
		(c) Consider use of a flow diagram	
Descriptive data	14*	(a) Give characteristics of study participants (eg demographic, clinical, social) and information on exposures and potential confounders	8
		(b) Indicate number of participants with missing data for each variable of interest	8
Outcome data	15*	Report numbers of outcome events or summary measures	8-9
Main results	16	(*a*) Give unadjusted estimates and, if applicable, confounder-adjusted estimates and their precision (eg, 95% confidence interval). Make clear which confounders were adjusted for and why they were included	10
		(*b*) Report category boundaries when continuous variables were categorized	
		(*c*) If relevant, consider translating estimates of relative risk into absolute risk for a meaningful time period	
Other analyses	17	Report other analyses done—eg analyses of subgroups and interactions, and sensitivity analyses	10-11
**Discussion**			
Key results	18	Summarise key results with reference to study objectives	12
Limitations	19	Discuss limitations of the study, taking into account sources of potential bias or imprecision. Discuss both direction and magnitude of any potential bias	12
Interpretation	20	Give a cautious overall interpretation of results considering objectives, limitations, multiplicity of analyses, results from similar studies, and other relevant evidence	12-14
Generalisability	21	Discuss the generalisability (external validity) of the study results	12
**Other information**			
Funding	22	Give the source of funding and the role of the funders for the present study and, if applicable, for the original study on which the present article is based	19

*Give information separately for cases and controls in case-control studies and, if applicable, for exposed and unexposed groups in cohort and cross-sectional studies.

Note: An Explanation and Elaboration article discusses each checklist item and gives methodological background and published examples of transparent reporting. The STROBE checklist is best used in conjunction with this article (freely available on the Web sites of PLoS Medicine at http://www.plosmedicine.org/, Annals of Internal Medicine at http://www.annals.org/, and Epidemiology at http://www.epidem.com/). Information on the STROBE Initiative is available at www.strobe-statement.org.

For peer review only
